# Supplementary material for: A two-level staging system for the embryonic morphogenesis of the Mediterranean fruit fly (medfly) Ceratitis capitata
Source: PLoS One. 2024 Dec 30;19(12):e0316391. doi: 10.1371/journal.pone.0316391 (PMC11684674; doi:10.1371/journal.pone.0316391)
Supplement: S1 Table — The entries are ordered primarily by phylogenetic distance to Ceratitis capitata and secondarily alphabetically. (DOCX) [file pone.0316391.s001.docx]

**S1 Table**

| **binominal name** | **common name** | **reference** | **techniques used** | **embryogenetic events** | | | | | |
| --- | --- | --- | --- | --- | --- | --- | --- | --- | --- |
| *Ceratitis capitata*  (Diptera: Tephritidae)  holometabolous | Mediterranean fruit fly, medfly | this study | fluorescence live imaging | **I** | **II** | **III** | **IV** | **V** | **VI** |
|  |  |  |  | staging system (17) | | | | | |
| *Dacus tryoni*  (Diptera: Tephritidae)  holometabolous | Queensland fruit fly | [1] | manual observation of development, serial sections | **I** | **II** | **III** | **IV** | **V** | **VI** |
|  |  |  |  | no staging | | | | | |
| *Drosophila melanogaster*  (Diptera: Drosophilidae)  holometabolous | fruit fly | [2] | non-fluorescence live imaging; fixation, mechanical sectioning and staining with non-fluorescent dyes | **I** | **II** | **III** | **IV** | **V** | **VI** |
|  |  |  |  | staging system (17) | | | | | |
| *Megaselia abdita*  (Diptera: Phoridae)  holometabolous | scuttle fly | [3] | non-fluorescence live imaging, fixation and staining with fluorescent dyes followed by widefield microscopy | **I** | **II** | **III** | **IV** | **V** | **VI** |
|  |  |  |  | staging system (17) | | | | | |
| *Aedes aegypti*  (Diptera: Culcidae)  holometabolous | yellow fever mosquito | [4,5] | fixation, mechanical sectioning and staining with non-fluorescent dyes | **I** | **II** | **III** | **IV** | **V** | **VI** |
|  |  |  |  | undergoes blastokinesis;  staging system (26, time points) | | | | | |
| *Aphidoletes aphidimyza*  (Diptera: Cecidomyiidae)  holometabolous | predatory gall midge | [6] | non-fluorescence live imaging | **I** | **II** | **III** | **IV** | **V** | **VI** |
|  |  |  |  | staging system (10) | | | | | |
| *Clogmia albipunctata*  (Diptera: Psychodidae)  holometabolous | moth midge, drain fly | [7] | non-fluorescence live imaging, fixation and staining with fluorescent dyes followed by widefield microscopy | **I** | **II** | **III** | **IV** | **V** | **VI** |
|  |  |  |  | staging system (17) | | | | | |
| *Nemotaulius admorsus*  (Trichoptera: Limnephilidae)  holometabolous | caddis fly | [8] | fixation, mechanical sectioning and staining with non-fluorescent dyes, scanning electron microscopy | **I** | **II** | **III** | **IV** | **V** | **VI** |
|  |  |  |  | staging system (10) | | | | | |
| *Philanisus plebeius*  (Trichoptera: Chathamidae)  holometabolous | marine caddis fly | [9] | fixation and non-stained observation | **I** | **II** | **III** | **IV** | **V** | **VI** |
|  |  |  |  | undergoes blastokinesis,  no staging system | | | | | |
| *Bombyx mori*  (Lepidoptera: Bombycidae)  holometabolous | domestic silkworm | [10] | fixation and staining with fluorescent dyes followed by confocal microscopy | **I** | **II** | **III** | **?** | **?** | **?** |
|  |  |  |  | no staging system | | | | | |
| *Endoclyta signifier* and  *Endoclyta excrescens*  (Lepidoptera: Hepialodae)  holometabolous | primitive moth | [11] | fixation, mechanical sectioning and staining with non-fluorescent dyes | **I** | **II** | **?** | **?** | **?** | **?** |
|  |  |  |  | no staging system | | | | | |
| *Nemophora albiantennella*  (Lepidoptera:Adelidae)  holometabolous | fairy moth | [12] | fixation and non-stained observation; fixation, mechanical sectioning and staining with non-fluorescent dyes; scanning electron microscopy | **I** | **II** | **III** | **IV** | **V** | **VI** |
|  |  |  |  | staging system (12) | | | | | |
| *Neomicropteryx nipponensis*  (Lepidoptera: Micropterygidae)  holometabolous | primitive moth | [13] | fixation, mechanical sectioning and staining with non-fluorescent dyes | **I** | **II** | **III** | **IV** | **V** | **VI** |
|  |  |  |  | staging system (14) | | | | | |
| *Rhagophthalmus ohbai*  (Coleoptera: Rhagophthalmidae)  holometabolous | glowworm | [14] | fixation, mechanical sectioning and staining with non-fluorescent dyes; scanning electron microscopy | **I** | **II** | **III** | **?** | **?** | **?** |
|  |  |  |  | staging system (8, not numbered) | | | | | |
| *Tribolium castaneum*  (Coleoptera: Tenebrionidae)  holometabolous | red flour beetle | [15] | light sheet-based fluorescence microscopy | **I** | **II** | **III** | **IV** | **V** | **VI** |
|  |  |  |  | no staging system | | | | | |
| *Xyleborus ferrugineus*  (Coleoptera: Scolytidae)  holometabolous | ambrosia beetle | [16,17] | non-fluorescence live imaging | **I** | **II** | **III** | **IV** | **V** | **VI** |
|  |  |  |  | staging system (12, time points) | | | | | |
| *Apis mellifera*  (Hymenoptera:Apidae)  holometabolous | honeybee | [18–20] | scanning electron microscopy | **I** | **II** | **-** | **-** | **V** | **VI** |
|  |  |  |  | staging system (6) | | | | | |
| *Nasonia vitripennis*  (Hymenoptera: Pteromalodae)  holometabolous | jewel wasp | [21] | non-fluorescence live imaging; fixation and staining with non-fluorescent dyes | **I** | **II** | **III** | **IV** | **V** | **VI** |
|  |  |  |  | staging system (8) | | | | | |
| *Acyrthosiphon pisum*  (Hemiptera: Aphidiae)  hemimetabolous | pea aphid | [22] | fixation and staining with fluorescent dyes/antibodies followed by confocal microscopy | **I** | **II** | **III** | **IV** | **?** | **VI** |
|  |  |  |  | undergoes blastokinesis;  staging system (20) | | | | | |
| *Galloisiana yuasai*  (Grylloblattoidea: Grylloblattidae)  hemimetabolous | - | [23] | fixation and staining with fluorescent dyes followed by wide field microscopy; fixation, mechanical sectioning and staining with non-fluorescent dyes; scanning electron microscopy; transmission electron microscopy | **I** | **II** | **III** | **-** | **V** | **VI** |
|  |  |  |  | undergoes blastokinesis;  staging system (11) | | | | | |
| *Gryllus bimaculatus*  (Orthoptera: Gryllidae)  hemimetabolous | two-spotted cricket | [24] | non-fluorescence live imaging, fixation and non-stained observation, fixation, germband preparation and staining with fluorescent dyes followed by confocal microscopy | **I** | **II** | **III** | **-** | **V** | **VI** |
|  |  |  |  | undergoes blastokinesis,  staging system (23) | | | | | |
| *Kamimuria tibialis*  (Plecoptera: Perlidae)  hemimetablous | stonefly | [25] | fixation, mechanical sectioning and staining with non-fluorescent dyes; scanning electron microscopy | **I** | **II** | **III** | **-** | **V** | **VI** |
|  |  |  |  | undergoes blastokinesis,  staging system (12) | | | | | |
| *Rhodnius prolixus*  (Hemiptera: Redivioidae)  hemimetabolous | kissing bug | [26] | fixation and staining with fluorescent dyes followed by wide field microscopy; fixation, mechanical sectioning and staining with non-fluorescent dyes; scanning electron microscopy; transmission electron microscopy | **I** | **II** | **III** | **-** | **IV** | **VI** |
|  |  |  |  | undergoes blastokinesis,  staging system (4, not numbered) | | | | | |
| *Ephemera japonica*  (Ephemeroptera: Ephemeridae)  hemimetabolous | mayfly | [27,28] | fixation, mechanical sectioning and staining with non-fluorescent dyes; scanning electron microscopy | **I** | **II** | **III** | **-** | **V** | **VI** |
|  |  |  |  | undergoes blastokinesis,  staging system (13) | | | | | |

**References**

1. Anderson DT. The Embryology of *Dacus tryoni* (Frogg.) [Diptera, Trypetidae (=Tephritidae)], the Queensland Fruit-Fly . Development. 1962. doi:10.1242/dev.10.3.248

2. Campos-Ortega JA, Hartenstein V. The Embryonic Development of *Drosophila melanogaster*. 2^nd^ Edition. Springer Berlin, Heidelberg. 1997. doi:10.1007/978-3-662-22489-2

3. Wotton KR, Jiménez-Guri E, García Matheu B, Jaeger J. A Staging Scheme for the Development of the Scuttle Fly *Megaselia abdita*. PLoS One. 2014. doi:10.1371/journal.pone.0084421

4. Raminani, L. N.; Cupp EW. Early embryology of *Aedes aegypti* (L.) (Diptera: Culicidae). Int J Insect Morphol Embryol. 1975. 10.1016/0020-7322(75)90028-8

5. Raminani LN, Cupp EW. Embryology of *Aedes aegypti* (L.) (Diptera: Culicidae): Organogenesis. Int J Insect Morphol Embryol. 1978. doi:10.1016/0020-7322(78)90009-0

6. Havelka J, Landa V, Landa V. Embryogenesis of *Aphidoletes aphidimyza* (Diptera: Cecidomyiidae): Morphological markers for staging of living embryos. Eur J Entomol. 2007. doi:10.14411/eje.2007.013

7. Jiménez-Guri E, Wotton KR, Gavilán B, Jaeger J. A Staging Scheme for the Development of the Moth Midge *Clogmia albipunctata*. PLoS One. 2014. doi:10.1371/journal.pone.0084422

8. Kobayashi Y, Ando H. Early embryonic development and external features of developing embryos of the caddisfly, *Nemotaulius admorsus* (Trichoptera: Limnephilidae). J Morphol. 1990. doi:10.1002/jmor.1052030108

9. Anderson DT, Lawson-Kerr C. The embryonic development of the marine caddis fly, *Philanisus plebeius* Walker (Trichoptera: Chathamidae). Biol Bull. 1977. doi:10.2307/1540693

10. Nagy L, Riddiford L, Kiguchi K. Morphogenesis in the Early Embryo of the Lepidopteran *Bombyx mori*. Dev Biol. 1994. doi:10.1006/dbio.1994.1241

11. Ando H, Tanaka M. Early embryonic development of the primitive moths, *Endoclyta signifer* Walker and *E. excrescens* Butler (Lepidoptera : Hepialidae). Int J Insect Morphol Embryol. 1980. doi:10.1016/0020-7322(80)90037-9

12. Kobayashi Y. Embryogenesis of the fairy moth, *Nemophora albiantennella* Issiki (Lepidoptera, Adelidae), with special emphasis on its phylogenetic implications. Int J Insect Morphol Embryol. 1998. doi:10.1016/S0020-7322(98)00006-3

13. Kobayashi Y, Ando H. The early embryonic development of the primitive moth, *Neomicropteryx nipponensis* lssiki (Lepidoptera, Micropterygidae). J Morphol. 1982. doi:10.1002/jmor.1051720302

14. Kobayashi Y, Suzuki H, Ohba N. Embryogenesis of the glowworm *Rhagophthalmus ohbai* Wittmer (Insecta: Coleoptera, Rhagophthalmidae), with emphasis on the germ rudiment formation. J Morphol. 2002. doi:10.1002/jmor.1109

15. Strobl F, Stelzer EHK. Non-invasive long-term fluorescence live imaging of *Tribolium castaneum* embryos. Development. 2014. doi:10.1242/dev.112706

16. Beeman SL, Norris DM. Embryogenesis of *Xyleborus ferrugineus* (Fabr.) (Coleoptera, Scolytidae). I. External morphogenesis of male and female embryos. J Morphol. 1977. doi:10.1002/jmor.1051520205

17. Beeman SL, Norris DM. Embryogenesis of *Xyleborus ferrugineus* (Fabr.) (Coleoptera, Scolytidae). II. Developmental rates of male and female embryos. J Morphol. 1977. doi:10.1002/jmor.1051520206

18. Fleig R, Sander K. Blastoderm development in honey bee embryogenesis as seen in the scanning electron microscope. Int J Invertebr Reprod Dev. 1985. doi:10.1080/01688170.1985.10510156

19. Fleig R, Sander K. Embryogenesis of the honeybee *Apis mellifera* L. (Hymenoptera: Apidae): An SEM study. Int J Insect Morphol Embryol. 1986. doi:10.1016/0020-7322(86)90037-1

20. Fleig R, Sander K. Honeybee morphogenesis: embryonic cell movements that shape the larval body. Development. 1988. doi:10.1242/dev.103.3.525

21. Bull AL. Stages of living embryos in the jewel wasp *Mormoniella* (*Nasonia*) *vitripennis* (Walker) (Hymenoptera: Pteromalidae). Int J Insect Morphol Embryol. 1982. doi:10.1016/0020-7322(82)90034-4

22. Miura T, Braendle C, Shingleton A, Sisk G, Kambhampati S, Stern DL. A comparison of parthenogenetic and sexual embryogenesis of the pea aphid *Acyrthosiphon pisum* (Hemiptera: Aphidoidea). J Exp Zool B Mol Dev Evol. 2003. doi:10.1002/jez.b.00003

23. Uchifune T, Machida R. Embryonic development of *Galloisiana yuasai* Asahina, with special reference to external morphology (Insecta: Grylloblattodea). J Morphol. 2005. doi:10.1002/jmor.10373

24. Donoughe S, Extavour CG. Embryonic development of the cricket *Gryllus bimaculatus*. Dev Biol. 2015. doi:10.1016/j.ydbio.2015.04.009

25. Kishimoto T, Ando H. External features of the developing embryo of the stonefly, *Kamimuria tibialis* (Pictet) (Plecoptera, Perlidae). J Morphol. 1985. doi:10.1002/jmor.1051830308

26. Kelly GM, Huebner E. Embryonic development of the hemipteran insect *Rhodnius prolixus*. J Morphol. 1989. doi:10.1002/jmor.1051990205

27. Tojo K, Machida R. Embryogenesis of the mayfly *Ephemera japonica* McLachlan (Insecta: Ephemeroptera, Ephemeridae), with special reference to abdominal formation. J Morphol. 1997. doi:10.1002/(SICI)1097-4687(199710)234:1<97::AID-JMOR9>3.0.CO;2-K

28. Tojo K, Machida R. Early embryonic development of the mayfly *Ephemera japonica* McLachlan (Insecta: Ephemeroptera, Ephemeridae). J Morphol. 1998. doi:10.1002/(SICI)1097-4687(199812)238:3<327::AID-JMOR4>3.0.CO;2-J
