# Supplementary material for: A two-level staging system for the embryonic morphogenesis of the Mediterranean fruit fly (medfly) Ceratitis capitata
Source: PLoS One. 2024 Dec 30;19(12):e0316391. doi: 10.1371/journal.pone.0316391 (PMC11684674; doi:10.1371/journal.pone.0316391)
Supplement: S2 Table — Primary stage identifiers are shown in bold. Only time points with clearly identifiable morphogenic changes compared to previous stages are listed. Except for cellularization, we only describe structures and processes for which our data provides morphological and/or dynamic evidence. In the ‘developmental process’ column, indentions indicate associated processes while parentheses refer to canonical processes summarized in S3 Table. In the ‘figures’ column, parentheses refer to figures that show the respective process secondarily while curly brackets refer to arrows in the respective figures. (DOCX) [file pone.0316391.s002.docx]

**S2 Table**

| **stage** | **time** | **%** | **developmental process** | **figure(s)** |
| --- | --- | --- | --- | --- |
| **I – blastoderm formation – stages 1 to 5 (5 total) – 00:00 h to 11:30 h (11:30 h total) – 0.0% to 18.4% (18.4% total) – Figure 2** | | | | |
| **I – 1** | **00:00 h** | **0.0%** | **pronuclear fusion results in the *zygote*, which is located in the inner regions of the *yolk*** | - |
| **I – 2** | **02:00 h** | **3.2%** | **the *yolk* begins withdrawing from both poles (*repeated withdrawal*)** | 2A, C; (1C) |
|  | 02:30 h | 4.0% | the majority of *zygotic nuclei* begin protruding towards the surface (*peripheral migration*) | 2A, ③ |
| **I – 3** | **03:30 h** | **5.6%** | **the *yolk* withdrawal at both poles reaches a relative maximum and starts reversing (*repeated withdrawal*)** | 2A, C; (1C, ①②) |
|  |  |  | the first *zygotic nuclei* reach the surface (*peripheral migration*) | 2A, ③ |
|  |  |  | the *zygotic nuclei* begin with the 10^th^ synchronous nuclear division | 2A |
|  | 04:00 h | 6.4% | the *zygotic nuclei* complete the 10^th^ and begin with the 11^th^ synchronous nuclear division | 2A |
|  |  |  | the *pole buds* emerge at the posterior tip of the *zygote* from the *zygotic nuclei* (*germ cell dynamics*) | 2A, ②, C |
| **I – 4** | **04:30 h** | **7.2%** | **the last *zygotic nuclei* reach the surface (end of *peripheral migration*)** | 2B |
|  |  |  | the *zygotic nuclei* complete the 11^th^ synchronous nuclear division and turn into the *blastoderm nuclei* | 2B |
|  |  |  | the *blastoderm nuclei* begin with the 12^th^ synchronous nuclear division | 2B, D, E |
|  |  |  | the *zygote* turns into the *syncytial blastoderm* (end of *peripheral migration*) | 2B |
|  | 05:00 h | 8.0% | the *blastoderm nuclei* complete the 12^th^ synchronous nuclear division | 2B, D, E |
|  |  |  | the *pole cells* derive at the posterior tip of the *syncytial blastoderm* from the *pole buds* (*germ cell dynamics*) | 2B, F, G |
|  | 05:30 h | 8.8% | the *blastoderm nuclei* begin with the 13^th^ synchronous nuclear division | 2B, D, E |
|  |  |  | the *pole cells* begin with their asynchronous mitotic division (*germ cell dynamics*) | 2B, ①, F |
|  | 06:00 h | 9.6% | the *blastoderm nuclei* complete the 13^th^ synchronous nuclear division | 2B, D, E |
|  |  |  | the *pole cells* complete their synchronous nuclear division (*germ cell dynamics*) | 2B ①, F |
|  |  |  | the *yolk* withdrawal at both poles completely reverses (*repeated withdrawal*) | 2B; (1C ②) |
| **I – 5** | **06:30 h** | **10.4%** | **the *blastoderm nuclei* complete the 13^th^ synchronous nuclear division** | 2B, D, E |
|  |  |  | the *blastoderm nuclei* probably begin with the synthesis of cell membranes (*cellularization*) | 2B; 3A |
|  | 11:00 h | 17.6% | the medio-ventral *blastoderm nuclei* exhibit a slight wavy appearance | 3A |
| **II – early gastrulation – stages 6 to 7 (2 total) – 11:30 h to 14:30 h (03:00 h total) – 18.4% to 23.2% (4.8% total) – Figure 3** | | | | |
| **II – 6** | **11:30 h** | **18.4%** | **the *blastoderm nuclei* probably complete the synthesis of cell membranes (end of *cellularization*)** | 3A |
|  |  |  | the *syncytial blastoderm* turns into the *cellular blastoderm* (end of *cellularization*) | 3A |
|  |  |  | the *yolk* turns into the *yolk sac* (end of *cellularization*) | 3A |
|  |  |  | the longitudinal *ventral furrow* arises medio-ventrally in the *cellular blastoderm* | 3A ①②, E ②③ |
|  | 12:30 h | 20.0% | the *cellular blastoderm* starts withdrawing from the anterior pole (*repeated withdrawal*) | 3B, (E); (1C ③) |
|  |  |  | the *ventral furrow* starts extending towards both tips of the *cellular blastoderm* | 3E ②③ |
|  |  |  | the transversal, antero-ventral / postero-dorsal slightly tilted *cephalic furrow* arises anteromedially in the *cellular blastoderm* | 3B ①, E |
|  | 13:00 h | 20.8% | the *ventral furrow* completes extending towards the anterior pole of the *cellular blastoderm* | 3E ② |
|  |  |  | the *cellular blastoderm* starts withdrawing from the posterior pole (*repeated withdrawal*) | 3B, (E); (1C ③) |
|  |  |  | the *posterior plate*, to which the *pole cells* adhere, emerges at the posterior tip of the *cellular blastoderm* (*germ cell dynamics*) | 3B, (E) |
| **II – 7** | **13:30 h** | **21.6%** | **the *anterior midgut primordium* emerges and invaginates at the anterior tip of the *ventral furrow* (*germ layer specification*)** | 3C, E, F |
|  |  |  | the *cellular blastoderm* differentiates into | 3C, D, (E, G) |
|  |  |  | the anterior *head*, the medio-ventral *thorax*, and the posterior *abdomen*, which constitute the *embryo* | as above |
|  |  |  | the medio-dorsal *amnioserosa*, which begins unfolding (*extra-embryonic membrane folding*) | as above |
|  |  |  | the *cephalic furrow* deepens laterally | 3C, E ①, (F, G) |
|  |  |  | the *ventral furrow* invaginates along the *thorax* and continues extending towards the posterior tip of the *abdomen* | 3C ①, E ②③, F ① |
|  |  |  | the *small and large lateral cell stripes* at the *head* begin with mitotic division^1^ | 3C, (D), G |
|  | 14:00 h | 22.4% | the *head* withdrawal from the anterior pole reaches a relative maximum and begins reversing (*repeated withdrawal*) | 3C; (1C) |
|  |  |  | the *small and large lateral cell stripes* complete mitotic division^1^ | 3C ②③, (D), G |
|  |  |  | the areas around the *small and large transversal cell stripes* at the *head* begin mitotic division^2^ | 3C ②③, (D), G |
|  |  |  | - the *head* remodels into | 3C, E, F, G |
|  |  |  | the antero-ventral / dorsal non-metameric lobe-like *procephalon* | as above |
|  |  |  | the postero-ventral *gnathocephalon*, which constitutes together with the *thorax* and the *abdomen* the *germband* | as above |
|  |  |  | - the *amnioserosa* completes unfolding and covers the *embryo* and the *yolk sac* medio-dorsally (*extra-embryonic membrane folding*) | 3C, D, (E) |
|  |  |  | - the *ventral furrow* closes along the already invaginated *thorax* / subsequently invaginating *abdomen* and differentiates into | 3C, E, F |
|  |  |  | the superficial *ectodermal layer* (*germ layer specification*) | as above |
|  |  |  | the internal *mesodermal layer* (*germ layer specification*) | as above |
|  |  |  | - the *posterior plate*, to which the *pole cells* adhere, turns into the *dorsal plate* (*germ cell dynamics*) | 3C ④, (D, E, G), H |
| **III – germband elongation – stages 8 to 11 (4 total) – 14:30 h to 24:30 h (10:00 h total) – 23.2% to 39.2% (16.0% total) – Figure 4** | | | | |
| **III – 8** | **14:30 h** | **23.2%** | - **the posterior tip of the *germband* begins anteriad elongation along the dorsal side towards with high speed** | 4A, D, E, H; (3E) |
|  |  |  | - - the *amnioserosa* begins folding (*extra-embryonic membrane folding*) | 4A, H |
|  |  |  | - - the *yolk sac* begins migration from the medio-dorsal surface to the internal region | 4A, H |
|  |  |  | - the *stomodeal cell plate* emerges antero-ventrally from the *procephalon* | 4A ①, F; (3E) |
|  |  |  | - the *anterior midgut primordium* internalizes (*germ layer specification*) | 4A ④; (3E, F) |
|  |  |  | - the areas around the *small and large transversal cell stripes* complete mitotic division^2^ | 4A; (3E, G) |
|  |  |  | - - all remaining ventrally and laterally located cells of the *procephalon* begin mitotic division | 4A; (3D, E, G) |
|  |  |  | - the *optic lobe primordium* emerges dorsally from the *procephalon* | 4A; (3D) |
|  | 15:00 h | 24.0% | - the *head* withdrawal reverses completely (*repeated withdrawal*) | 4A; (1C {4}) |
|  |  |  | - all remaining ventrally and laterally located cells of the *procephalon* complete mitotic division | 4A; (3D, E) |
|  |  |  | - the *dorsal plate*, to which the *pole cells* adhere, internalizes, and turns into the *posterior midgut primordium* (end of *germ cell dynamics* and end of *germ layer specification*) | 4A, C ①②, H, (J) |
|  |  |  | - the *stomodeal cell plate* sinks inwards and turns into the *stomodeal invagination* | 4A ②, F; (3E, F) |
|  | 15:30 h | 24.8% | - the *optic lobe primordium* begins with mitotic divisions | 4A, J |
|  |  |  | - the *stomodeal invagination* and the *anterior midgut primordium* connect | 4F ① |
|  |  |  | - the *amnioproctodeal invagination* emerges at the posterior tip of the *ventral furrow* and around the invaginating *posterior midgut primordium* from the *ectodermal layer* | 4A ⑤ |
|  | 16:00 h | 25.6% | - the *ectodermal layer* differentiates into | 4A, (C), G |
|  |  |  | the *ventral epidermal primordium* | as above |
|  |  |  | the *lateral epidermal primordia* | as above |
|  |  |  | - the *abdomen* withdrawal from the posterior pole reaches a momentary standstill (*repeated withdrawal*) | 4A; (1C) |
| **III – 9** | **16:30 h** | **26.4%** | - **the posterior tip of the *germband* continues anteriad elongation along the dorsal side with moderate speed** | 4A, D, E, H, J |
|  |  |  | - the *stomodeal invagination* turns into the *stomodeum*, which becomes the anteromedial part of the *digestive tract* (*digestive system formation*) | 4A ③, F |
|  |  |  | - the *cephalic furrow* levels out | 4A, G |
|  |  |  | - the *ventral furrow* levels out | 4A, (H, I) |
|  |  |  | - the first *neuroblasts* begin segregating from the *ventral epidermal primordium* | 4G ①②③ |
|  |  |  | - the *amnioproctodeal invagination* differentiates into | 4A, C, (H) |
|  |  |  | the *proctodeum*, which starts internalizing and becomes the posteromedial part of the *digestive tract* (*digestive system formation*) | as above |
|  |  |  | the *proctodeal opening*, which becomes the posterior part of the *digestive tract* (*digestive system formation*) | as above |
|  | 17:00 h | 27.2% | - the *proctodeum* completes internalizing | 4A, H |
|  | 17:30 h | 28.0% | - the *proctodeal opening* changes from a dorsal orientation to an anterior orientation | 4A ⑥, H, (J) |
| **III – 10** | **18:00 h** | **28.8%** | - **the posterior tip of the *germband* continues anteriad elongation along the dorsal side with low speed** | 4B, D, E |
|  | 19:00 h | 30.4% | - the *ventral mesectoderm* emerges ventrally along the *thorax* and *abdomen* from the *ventral epidermal primordium* | 4I, (J) |
|  |  |  | - the *head* begins withdrawing from the anterior pole (*repeated withdrawal*) | 4B, J, I; (1C ⑤) |
|  | 21:00 h | 33.6% | - the *optic lobe primordium* completes mitotic divisions | 4B, J, (I) |
| **III – 11** | **22:00 h** | **35.2%** | - **the posterior tip of the *germband* completes anteriad elongation and remains in a quiescent position** | 4B |
|  |  |  | - - the *amnioserosa* completes folding (*extra-embryonic membrane folding*) | 4B, (H, J, I) |
|  |  |  | - - the *yolk sac* completes migration from the medio-dorsal surface to the interior regions | 4B, (H, J, I) |
|  |  |  | - the *head* withdrawal from the anterior pole reaches a momentary standstill (*repeated withdrawal*) | 4B; (1C ⑥) |
|  |  |  | - the *abdomen* continues withdrawing after the momentary standstill (*repeated withdrawal*) | 4B; (1C ⑥) |
|  | 24:00 h | 38.4% | - the *gnathal protuberances* emerge from the *gnathocephalon* (*metamerization*) | 4B |
| **IV – germband retraction – stage 12 (1 total) – 24:30 h to 32:00 h (07:30 h total) – 39.2% to 51.2% (12.0% total) – Figure 5** | | | | |
| **IV – 12** | **24:30 h** | **39.2%** | - **the posterior tip of the *germband* begins posteriad retraction along the dorsal side** | 5A |
|  |  |  | - - the *amnioserosa* begins unfolding again (*extra-embryonic membrane folding*) | 5A |
|  |  |  | - - the *yolk sac* begins protruding towards the medio-dorsal surface | 5A |
|  | 26:00 h | 41.6% | - the *dorsal epidermal primordia* emerge laterally from the *lateral epidermal primordia* | 5A, B ① |
|  | 26:30 h | 42.4% | - the *clypeolabrum* emerges antero-dorsally from the *procephalon* | 5A, B |
|  | 27:30 h | 44.0% | - the *abdomen* withdrawal at the posterior pole reaches a relative maximum and begins reversing (*repeated withdrawal*) | 5A, B; (1C) |
|  | 28:30 h | 45.6% | - the *intersegmental grooves* arise and separate the *thorax* into three and the *abdomen* into nine segments (*metamerization*) | 5A, B ②③④ |
|  | 30:30 h | 48.8% | - the *clypeolabrum* becomes thinner and more pronounced | 5A, B |
|  |  |  | - the *proctodeal opening* becomes posterior-dorsally located | 5A ① |
|  | 31:30 h | 50.4% | - the *amnioserosa* completes unfolding and covers the *embryo* and *yolk sac* medio-dorsally (*extra-embryonic membrane folding*) | 5A |
|  |  |  | - - the *yolk sac* completes protruding towards the medio-dorsal surface | 5A |
| **V – dorsal closure – stages 13 to 15 (3 total) – 32:00 to 45:00 h (13:00 h total) – 51.2% to 72.0% (20.8% total) – Figure 6** | | | | |
| **V – 13** | **32:00 h** | **51.2%** | - **the posterior tip of the *germband* completes posteriad retracting** | 6A |
|  |  |  | - the *ventral epidermal primordium* and *lateral epidermal primordia* of the *thorax* begin anteriad migration (*head involution*) | 6A, (D) |
|  |  |  | - the *dorsal epidermal primordia* begin dorsolaterad migration over the *amnioserosa* (*dorsal zippering*) | 6A, (D), G |
|  |  |  | - - the *amnioserosa* starts folding again (*extra-embryonic membrane folding*) | 6A, (D), G |
|  | 33:30 h | 53.6% | - the *anterior dorsal gap* arises between the *procephalon* and the *amnioserosa* | 6A ①, C, D |
|  |  |  | - - the *dorsal folds* emerge dorsally from the *gnathocephalon* | 6D |
| **V – 14** | **34:30 h** | **55.2%** | - **the *clypeolabrum* turns from an antero-dorsal to an antero-ventral orientation (*head involution*)** | 6A, (D), F |
|  |  |  | - - the *head* continues withdrawing after the momentary standstill (*repeated withdrawal*) | 6A, C, D, F; (1C ⑦) |
|  |  |  | - - the *dorsal folds* fuse along the *anterior dorsal gap* and begin anteriad migration (*head involution*) | 6A, D |
|  |  |  | - the *dorsal epidermal primordia* begin with anteromedial and posteromedial fusion (*dorsal zippering*) | 6A, G ①② |
|  |  |  | - the *abdomen* withdrawal at the posterior pole reaches a relative minimum and begins re-reversing (*repeated withdrawal*) | 6A, D, G; (1C ⑦) |
|  | 35:00 h | 56.0% | - the *stomodeum* begins retracting (*head involution*) | 6A, C, E ③ |
|  |  |  | - the *gnathal protuberances* migrate anterior and differentiate into | 6A, C |
|  |  |  | the *mandibular buds* (*head involution*) | 6A ③, C ①, E ① |
|  |  |  | the *maxillary buds* (*head involution*) | 6A |
|  |  |  | the *labial buds* (*head involution*) | 6A |
|  | 36:30 h | 58.4% | - the *labial buds* migrate ventrally (*head involution*) | 6C |
|  |  |  | - the *midgut* originates from the fusion of the *anterior midgut primordium* and the *posterior midgut primordium*, which becomes the medial part of the *digestive tract* (*digestive system formation*) | 6A ②, (G) |
|  |  |  | - - the *amnioserosa* completes folding and begins internalizing together with the *yolk sac* into the *midgut* (*extra-embryonic membrane folding*) | 6A, (D), G |
|  | 37:00 h | 59.2% | - the *labial buds* fuse ventrally (*head involution*) | 6A ④, C ②, E ② |
| **V – 15** | **38:00 h** | **60.8%** | - **the *abdomen*** **withdrawal at the posterior pole reaches the absolute maximum (*repeated withdrawal*)** | 6A; (1C) |
|  |  |  | - - the posterior tip of the *germband* begins flipping into the posterior pole | 6A, B, (D) |
|  |  |  | - the *dorsal epidermal primordia* complete anteromedial and posteromedial fusion (*dorsal zippering*) | 6A, G ①② |
|  |  |  | - - the *dorsal epidermal primordia* begin with medial fusion (*dorsal zippering*) | 6A, G ③④ |
|  |  |  | - the *antennomaxillary complexes* originate from the fusion of the *maxillary buds* and the antero-ventral region of the *procephalon* (*head involution*) | 6A ⑤, C ③, E ④, F |
|  |  |  | - - the *clypeolabrum* begins retracting (*head involution*) | 6A, C, D, E, F, (G) |
|  |  |  | - - the *mandibular buds* begin retracting (*head involution*) | 6A, C ①, E ①, F |
|  |  |  | - the *anterior dorsal gap* levels out | 6A, C, (D) |
|  | 38:30 h | 61.6% | - the fused *labial buds* begin retracting (*head involution*) | 6B, C ②, E ② |
|  |  |  | - the *antennomaxillary complexes* begin anteriad migration (*head involution*) | 6B, E ④, F |
|  | 39:30 h | 63.2% | - the fused *dorsal folds* cover the *procephalon* and continue anteriad migration (*head involution*) | 6B, D, E ⑤ |
|  | 40:30 h | 64.8% | - the *head* withdrawal at the anterior pole reaches the absolute maximum and begin reversing (*repeated withdrawal*) | 6B, D; (1C ⑧) |
|  |  |  | - the *abdomen* withdrawal at the posterior pole begins reversing (*repeated withdrawal*) | 6B, D; (1C ⑧) |
|  |  |  | - the fused *dorsal folds* cover the retracting *clypeolabrum* and continue anteriad migration (*head involution*) | 6B, D |
|  | 41:30 h | 66.4% | - the *intersegmental grooves* of the *thorax* and the *abdomen* transiently level out (*metamerization*) | 6B |
|  | 43:30 h | 69.6% | - the *head* withdrawal at the anterior pole partially reverses (*repeated withdrawal*) | 6B, D; (1C ⑨) |
|  |  |  | - the *abdomen* withdrawal at the posterior pole partially reverses (*repeated withdrawal*) | 6B, D; (1C ⑨) |
|  | 44:00 h | 70.4% | - the *dorsal epidermal primordia* complete migration dorsolaterally over the *amnioserosa* (*dorsal zippering*) | 6D |
|  |  |  | - - the *amnioserosa* completes internalizing together with the *yolk sac* into the *midgut* (end of *extra-embryonic membrane folding*) | 6D |
|  | 44:30 h | 71.2% | - the *clypeolabrum*, the *stomodeum*, the fused *labial buds* and the *mandibular buds* complete retraction (*head involution*) | 6B |
|  |  |  | - the *antennomaxillary complexes* complete anteriad migration (*head involution*) | 6B |
|  |  |  | - the posterior tip of the *germband* completes flipping into the posterior pole | 6B |
|  |  |  | - - the *proctodeal opening* becomes posterior located | 6B |
| **VI – muscular movement – stages 16 to 17 (2 total) – 45:00 h to 62:30 h (17:30 h total) – 72.0% to 100.0% (28.0% total) – Figure 7** | | | | |
| **VI – 16** | **45:00 h** | **72.0%** | - **the *dorsal epidermal primordia* complete medial fusion and turn into the *dorsal epidermis* (end of *dorsal zippering*)** | 7A, (C) |
|  |  |  | - - the *ventral epidermal primordium* completes anteriad migration and turns into the *ventral epidermis* (end of *head involution*) | 7A, C |
|  |  |  | - - and *lateral epidermal primordia* complete anteriad migration and turn into the *lateral epidermis* (end of *head involution*) | 7A, C |
|  |  |  | - the *antennomaxillary complexes* fuse anterior (end of *head involution*) | 7A, C |
|  |  |  | - the *intersegmental grooves* arise again (end of *metamerization*) | 7A, (B), C |
|  |  |  | - the posterior tip of the *ventral cord*, which extends to the 8^th^ segment of the *abdomen*, begins shortening | 7A, B |
|  | 49:00 h | 78.4% | - the fused *dorsal folds* complete anteriad migration and turn into the *dorsal pouch* (end of *head involution*) | 7C |
|  |  |  | - - the *atrium* forms anteriorly through contribution of the *clypeolabrum*, the fused *antennomaxillary complexes*, the *mandibular buds,* and the fused *labial buds* (*digestive system formation*) | 7A, C |
|  |  |  | - - the *digestive tract* originates through consecutive connection of the *atrium*, the *stomodeum*, the *midgut*, the *proctodeum* and the *proctodeal opening* (end of *digestive system formation*) | 7A, C |
|  |  |  | - the posterior tip of the *ventral cord* shortens to the 7^th^ segment of the *abdomen* | 7A, C |
|  | 52:00 h | 83.2% | - the posterior tip of the *ventral cord* shortens to the 6^th^ segment of the *abdomen* | 7A, C |
| **VI – 17** | **54:30 h** | **87.2%** | - **the posterior tip of the *ventral cord* shortens to the 5^th^ segment of the *abdomen*** | 7A, B |
|  |  |  | - the *head* withdrawal at the anterior pole nearly completely reverses (end of *repeated withdrawal*) | 7A; (1C) |
|  |  |  | - the *abdomen* withdrawal at the posterior pole nearly completely reverses (end of *repeated withdrawal*) | 7A; (1C) |
|  | 56:00 h | 89.6% | - the *embryo* begins with regular muscular movement | 7B |
|  | 58:00 h | 92.8% | - the posterior tip of the *ventral cord* completes shortening between the 4^th^ and 5^th^ segment of the *abdomen* | 7C |
| **Beyond** | **62:30 h** | **100.0%** | - the *embryo* completes embryonic development, hatches, and turns into the larva | 7D |
