# Supplementary material for: A two-level staging system for the embryonic morphogenesis of the Mediterranean fruit fly (medfly) Ceratitis capitata
Source: PLoS One. 2024 Dec 30;19(12):e0316391. doi: 10.1371/journal.pone.0316391 (PMC11684674; doi:10.1371/journal.pone.0316391)
Supplement: S3 Table — In the ‘figures’ column, parentheses refer to figures that show the respective process secondarily. (DOCX) [file pone.0316391.s003.docx]

**S3 Table**

| **process** | **embryogenetic event** | | | | | | **description / rationale** | **figures** |
| --- | --- | --- | --- | --- | --- | --- | --- | --- |
| *repeated withdrawal* | **I** | **II** | **III** | **IV** | **V** | **VI** | During the entire embryogenesis, first the *yolk*, later the *head* and the *abdomen*, withdraw and reverse multiple times from the anterior and posterior pole. Withdrawal from both poles occurs sometimes synchronously and sometimes independently from each other, which leads to a non-trivial embryo length-over-time graph (Figure 1C). In some cases, withdrawal is linked certain processes, for example emergence of the *posterior plate* during stage 6 (Figure 1C, ③) or the movement of the *clypeolabrum* during stage 14 (Figure 1C, ⑦). Withdrawal of both poles reaches an absolute maximum of about 5% of total length for both the *head* at the anterior and the *abdomen* posterior pole in parallel during stage 15 (Figure 1C, ⑧). Withdrawal *per se* is not a unique feature of medfly development, but happens also in other dipterans [1–3] and in more distantly related species such as the red flour beetle [4,5] or the two-spotted cricket [6]. However, the resulting length-over-time graph (Figure 1C) is most probably unique and thus serves as a characteristic curve for the medfly. | 1C; 2A, B, C; 3B, C, (E); 4A, B, J, I; 5A, B; 6A, B, C, D, F, G; 7A |
| *peripheral migration* | **I** | **-** | **-** | **-** | **-** | **-** | After nine synchronous nuclear divisions within the inner regions of the *yolk*, respectively allocated *zygotic nuclei* start protruding to the surface during stage 2 (Figure 2A, ③) and immediately proceed through the 10^th^ and 11^th^ synchronous nuclear division. Similar to the development of other insect species [1,7], a certain fraction of nuclei remain in the inner regions and become vitellophages (S5 Table). Once completed, the *zygotic nuclei*, which have a relatively large size, turn into the *blastoderm nuclei*, and the *zygote* turns into the *syncytial blastoderm*. The general procedure of several synchronous nuclear divisions followed by outward migration is a fundamental feature of insect development, but the quantitative frame may differ between species of different orders (S1 Table). | 2A, B |
| *germ cell dynamics* | **I** | **II** | **III** | **-** | **-** | **-** | During stage 3, several zygotic nuclei give rise to the *pole buds* at the posterior tip of the *zygote* (Figure 2A, ②). Slightly later, during stage 4, the *pole buds* pinch off and turn into the *pole cells*, which undergo synchronous nuclear division (Figure 2B, ①). During stage 6, the *pole cells* adhere to the emerging *posterior plate*, which turns into the *dorsal plate* during stage 7 (Figure 3C). During stages 8, the *pole cells* stay attached to the *posterior midgut primordium* which derived from the *dorsal plate*. Thus, they migrate with the posterior tip of the anteriad elongating *germband* along the dorsal side (Figure 4A, ③, and C, ①). The *pole cells* fade from sight as the *posterior midgut primordium* invaginates and the *proctodeal opening* changes to an anterior orientation, and their further development cannot be followed anymore (Figure 4A, ④). They remain with the *posterior midgut primordium* for some more time before formation of gonads take place [8] and the *pole cells* turn into germ cells. The process in very similar to the fruit fly [1,9] and the scuttle fly [2], but in the moth midge [3], and the red flour beetle [5,7], pole cells are not properly visible during the early stages of development. | 2A, B, C, F, G; 3B, C, (D, E, G), H; 4A, C, H, (J) |
| *cellularization* | **I** | **II** | **-** | **-** | **-** | **-** | Stage 5 is a long period of morphogenic quiescence, since the *blastoderm nuclei* synthetize cell membranes during this time, where almost all changes occur on the sub-cellular level. In brief, the furrow growth is mediated by contracting microfilaments, and the membrane is enlarged by vesicle fusion [10]. Once completed, the *blastoderm nuclei* are individualized, the *syncytial blastoderm* has turned into the *cellular blastoderm*, and instead of nuclear divisions, mitotic divisions occur now. This process is also a fundamental feature of insect development (S1 Table), but a detailed comparison is not possible since no membrane marker is available for the medfly. | 2B; 3A |
| *germ layer specification* | **-** | **II** | **III** | **-** | **-** | **-** | The ectodermal, mesodermal and endodermal germ layers are allocated under participation of the *ventral furrow* and the *dorsal plate* during stages 6, 7 and 8. Once completed, the *anterior midgut primordium* (Figure 4A, ②), the *mesodermal layer*, the *ectodermal layer* and the *posterior midgut primordium* (Figure 4C, ②) have differentiated. This process is highly similar in closely related dipterans, for example the fruit fly [1] and the scuttle fly [2,11], but it is not yet clear if certain structures, such as the *ventral furrow*, also arise in more distantly related dipterans such as the moth midge [3]. In predatory gall midge, a sister species to the moth midge, no *ventral furrow* could be observed [12]. | 3C, E, F; 4A, C, H, (J) |
| *extra-embryonic membrane folding* | **-** | **II** | **III** | **IV** | **V** | **-** | The *amnioserosa* differentiates medio-dorsally from the *cellular blastoderm* at the beginning of stage 7 as the only extra-embryonic membrane. It immediately begins to unfold for the first time, covering the *embryo* and the *yolk sac* and folds throughout 8, 9, and 10 as the posterior tip of the *germband* anteriad elongates along the dorsal side and remains in the conformation during stage 11. During stage 12, it unfolds a second time as the posterior tip of the *germband* retracts. As the *dorsal zippering* process proceeds, the amnioserosa folds a second time and finally internalizes together with the *yolk sac* into the *midgut* during stage 15. The process of *amnioserosa* formation and rearrangement differs remarkably from the fruit fly [1,13–15] and is critically analyzed in the discussion. the scuttle fly [2,16–18], the moth midge [3] and more distantly related species such as *Tribolium* [19] have two extra-embryonic membranes and thus a completely different rearrangement concept. | 3C, D, (E), (G); 4A, B, H, (I, J); 6A, (D), G |
| *digestive system formation* | **-** | **-** | **III** | **IV** | **V** | **VI** | Formation of the *digestive tract* is one of the most comprehensive processes in the embryonic development of the medfly that involves mainly ectodermal and endodermal tissue that differentiates during the *germ layer specification* process*.* In brief, the digestive tract originates through consecutive connection of five structures: (i) the anterior *atrium*, which forms during stage 16, (ii) the anteromedial *stomodeum*, which derives from the *stomodeal invagination* at the beginning of stage 9, (iii) the medial *midgut*, which originates from the fusion of two endodermal primordia during stage 14 (Figure 6A, ③), (iv) the posteromedial *proctodeum*, which differentiates from the *amnioproctodeal invagination* at the beginning of stage 9, and the (v) posterior *proctodeal opening*, which also differentiates from the *amnioproctodeal invagination* at the beginning of stage 9. Due to limited resolution within inner regions of the *embryo*, the mentioned structures cannot not subdivided further as done for *the fruit fly* [1], for example the *stomodeum* is not subdivided into pharynx, esophagus and proventriculus. Formation of the *digestive tract* appears to be very similar to the fruit fly [1,20,21] and the scuttle fly [2], but for other insect species, not enough information for a proper comparison is available (S1 Table). | 4A, C, F, (H); 6A, (G); 7A, C; 5A; 6A, D, G |
| *metamerization* | **-** | **-** | **III** | **IV** | **V** | **VI** | During the later stages of embryonic development, the *germband* becomes subdivided into fifteen segments. Metameres can be recognized first during stage 11, when the *gnathal protuberances* emerge from the *gnathocephalon*, and later on also during stage 12, when the *intersegmental grooves* divide the *thorax* into three (Figure 5B, ②③④) and the *abdomen* into nine segments. The grooves disappear transiently during stage 15 but reappear at the beginning of stage 16. Due to limited resolution within the inner regions of the *embryo*, metamerism can only be confirmed for the ectoderm, but similar to the fruit fly [1], it can be reasonably assumed that the mesoderm, but not the endoderm is segmentally organized. The number of segments is identical to the fruit fly [1] and the scuttle fly [22]. | 5A, B; 6B; 7A, (B), C |
| *head involution* | **-** | **-** | **-** | **-** | **V** | **VI** | Involution of the *head* is a complex morphogenic process that occurs during stages 14, 15 and 16 and can be subdivided into three directed migrations: (i) the *ventral epidermal primordium*, the *lateral epidermal primordia* and the *antennomaxillary complexes* (Figure 6E, ④) migrate anteriad, (ii) the fused *dorsal folds* (Figure 6E, ⑤) cover the *procephalon* and the *clypeolabrum* and eventually turns into the *dorsal pouch*, and (iii) the *stomodeum* (Figure 6A, ③), the *clypeolabrum*, the *mandibular buds* (Figure 6C, ①, and E, ①) and the fused *labial buds* retract. Once head involution is completed, the retracted structures and the now fused *antennomaxillary complexes* have formed the *atrium*. Head involution proceeds highly similar compared to the fruit fly [1], it should however be noted that the c*lypeolabrum* is larger and more pronounced in the medfly. | 6A, B, C, D, E, F, (G); 7A, C |
| *dorsal zippering* | **-** | **-** | **-** | **-** | **V** | **VI** | At the end of stage 12, the *embryo* is covered dorsally only by the *amnioserosa*, an extra-embryonic membrane. During stage 13, 14 and 15, the bilateral *dorsal epidermal primordia* migrate dorsolaterally over the *amnioserosa* and begin fusing. Thereby, two leading edges emerge anteromedial and posteromedial that gradually approach each other (Figure 6G, ①②③④) and form a tight seam along the dorsal midline. Once completed, the *dorsal epidermal primordia* have turned into the *dorsal epidermis*, a continuous cell layer that seals the *embryo* dorsally. Zippering along the dorsal midline is a conserved process in insects and even in far more distantly related species such as vertebrates [23]. The process is very similar to the fruit fly [1,24] and moderately similar to the red flour beetle [5,25], since the red flour beetle generates and degrades two extra-embryonic membranes. | 6A, D, G; 7A, C |

**References**

1. Campos-Ortega JA, Hartenstein V. The Embryonic Development of *Drosophila melanogaster*. ^nd^ Edition. Springer Berlin, Heidelberg. 1997. doi:10.1007/978-3-662-22489-2

2. Wotton KR, Jiménez-Guri E, García Matheu B, Jaeger J. A Staging Scheme for the Development of the Scuttle Fly *Megaselia abdita*. PLoS One. 2014. doi:10.1371/journal.pone.0084421

3. Jiménez-Guri E, Wotton KR, Gavilán B, Jaeger J. A Staging Scheme for the Development of the Moth Midge *Clogmia albipunctata*. PLoS One. 2014. doi:10.1371/journal.pone.0084422

4. Benton MA, Akam M, Pavlopoulos A. Cell and tissue dynamics during *Tribolium* embryogenesis revealed by versatile fluorescence labeling approaches. Development. 2013. doi:10.1242/dev.096271

5. Strobl F, Stelzer EHK. Non-invasive long-term fluorescence live imaging of *Tribolium castaneum* embryos. Development. 2014. doi:10.1242/dev.112706

6. Donoughe S, Extavour CG. Embryonic development of the cricket *Gryllus bimaculatus*. Dev Biol. 2015. doi:10.1016/j.ydbio.2015.04.009

7. Handel K, Grünfelder CG, Roth S, Sander K. *Tribolium* embryogenesis: a SEM study of cell shapes and movements from blastoderm to serosal closure. Dev Genes Evol. 2000. doi:10.1007/s004270050301

8. Riparbelli MG, Callaini G, Dallai R. Primordial germ cell migration in the *Ceratitis capitata* embryo. Tissue Cell. 1996. doi:10.1016/S0040-8166(96)80048-6

9. Tripathy R, Kunwar PS, Sano H, Renault AD. Transcriptional regulation of *Drosophila* gonad formation. Dev Biol. 2014. doi:10.1016/j.ydbio.2014.05.026

10. Callaini G. Cleavage and membrane formation in the blastoderm of the dipteran *Ceratitis capitata* Wied. J Morphol. 1987. doi:10.1002/jmor.1051930308

11. Caroti F, Urbansky S, Wosch M, Lemke S. Germ line transformation and in vivo labeling of nuclei in Diptera: report on *Megaselia abdita* (Phoridae) and *Chironomus riparius* (Chironomidae). Dev Genes Evol. 2015. doi:10.1007/s00427-015-0504-5

12. Havelka J, Landa V, Landa V. Embryogenesis of *Aphidoletes aphidimyza* (Diptera: Cecidomyiidae): Morphological markers for staging of living embryos. Eur J Entomol. 2007. doi:10.14411/eje.2007.013

13. Lamka ML, Lipshitz HD. Role of the Amnioserosa in Germ Band Retraction of the *Drosophila melanogaster* Embryo. Dev Biol. 1999. doi:10.1006/dbio.1999.9409

14. Scuderi A, Letsou A. Amnioserosa is required for dorsal closure in *Drosophila*. Dev Dyn. 2005. doi:10.1002/dvdy.20306

15. Lacy ME, Hutson MS. Amnioserosa development and function in *Drosophila* embryogenesis: Critical mechanical roles for an extraembryonic tissue. Dev Dyn. 2016. doi:10.1002/dvdy.24395

16. Rafiqi AM, Lemke S, Ferguson S, Stauber M, Schmidt-Ott U. Evolutionary origin of the amnioserosa in cyclorrhaphan flies correlates with spatial and temporal expression changes of *zen*. Proc Natl Acad Sci U S A. 2008. doi:10.1073/pnas.0709145105

17. Rafiqi AM, Lemke S, Schmidt-Ott U. Postgastrular *zen* expression is required to develop distinct amniotic and serosal epithelia in the scuttle fly *Megaselia*. Dev Biol. 2010. doi:10.1016/j.ydbio.2010.01.040

18. Rafiqi AM, Park C-H, Kwan CW, Lemke S, Schmidt-Ott U. BMP-dependent serosa and amnion specification in the scuttle fly *Megaselia abdita*. Development. 2012. doi:10.1242/dev.083873

19. Schmidt-Ott U, Kwan CW. Morphogenetic functions of extraembryonic membranes in insects. Curr Opin Insect Sci. 2016. doi:10.1016/j.cois.2016.01.009

20. Buchon N, Osman D, David FPA, Yu Fang H, Boquete J-P, Deplancke B, et al. Morphological and Molecular Characterization of Adult Midgut Compartmentalization in *Drosophila*. Cell Rep. 2013. doi:10.1016/j.celrep.2013.04.001

21. Lemaitre B, Miguel-Aliaga I. The Digestive Tract of *Drosophila melanogaster*. Annu Rev Genet. 2013. doi:10.1146/annurev-genet-111212-133343

22. Stauber M, Taubert H, Schmidt-Ott U. Function of *bicoid* and *hunchback* homologs in the basal cyclorrhaphan fly *Megaselia* (Phoridae). Proc Natl Acad Sci U S A. 2000. doi:10.1073/pnas.190095397

23. Millard TH, Martin P. Dynamic analysis of filopodial interactions during the zippering phase of *Drosophila* dorsal closure. Development. 2008. doi:10.1242/dev.014001

24. Jacinto A, Woolner S, Martin P. Dynamic Analysis of Dorsal Closure in *Drosophila*: From Genetics to Cell Biology. Dev Cell. 2002. doi:10.1016/S1534-5807(02)00208-3

25. Panfilio KA, Oberhofer G, Roth S. High plasticity in epithelial morphogenesis during insect dorsal closure. Biol Open. 2013. doi:10.1242/bio.20136072
