# Supplementary material for: A two-level staging system for the embryonic morphogenesis of the Mediterranean fruit fly (medfly) Ceratitis capitata
Source: PLoS One. 2024 Dec 30;19(12):e0316391. doi: 10.1371/journal.pone.0316391 (PMC11684674; doi:10.1371/journal.pone.0316391)
Supplement: S4 Table — This glossary table provides a definition of all structural terms highlighted in italics throughout this publication. In the ‘figures’ column, parentheses refer to figures that show the respective process secondarily while curly brackets refer to arrows in the respective figures. (DOCX) [file pone.0316391.s004.docx]

**S4 Table**

| **structure** | **embryogenetic event** | | | | | | **description / rationale** | **figures** | **reference / comment** |
| --- | --- | --- | --- | --- | --- | --- | --- | --- | --- |
| *abdomen* | **-** | **II** | **III** | **IV** | **V** | **VI** | Large tissue region differentiating posteriorly from the *cellular blastoderm* at the beginning of stage 7. Constitutes together with the *head* and the *thorax* the *embryo*. Composes together with the *gnathocephalon* and the *thorax* the *germband* and becomes divided into nine segments during stage 12 as part of *metamerization* process. | 3C, D, (E), (F), (G); 4A, B, I, (J); 5A, B; 6A, B, D, G; 7A, B, C | Adopted from the fruit fly [1,2]. |
| *amnioproctodeal invagination* (transient) | **-** | **-** | **III** | **-** | **-** | **-** | Tubular structure emerging at the posterior tip of the *ventral furrow* and around the invaginating *posterior midgut primordium* from the *ectodermal layer* during stage 8. Differentiates into the *proctodeum* and the *proctodeal opening* at the beginning of stage 9. | 4A, C, (H) | Adopted from the fruit fly [2–4], the scuttle fly [5,6] and the moth midge [7]. The term ‘proctodeal invagination’ is used synonymously [8,9]. |
| *amnioserosa* (transient) | **-** | **II** | **III** | **IV** | **V** | **-** | Extra-embryonic membrane differentiating medio-dorsally from the *cellular blastoderm* at the beginning of stage 7. Unfolds and folds two times without any mitotic division during stages 7 to 14 as part of the *extra-embryonic membrane folding* process. Internalizes together with the *yolk sac* into the *midgut* during stage 15 after the *extra-embryonic membrane folding* process has ended. | 3C, D, (E, G); 4A, B, H, (J, I); 5A; 6A, D, G | Adopted from the fruit fly [2,4,10,11]. Common term for the extra-embryonic membrane in cyclorrhaphan flies [12]. |
| *antennomaxillary complexes* | **-** | **-** | **-** | **-** | **V** | **VI** | Bilateral structures originating antero-laterally from the fusion of the *maxillary buds* and the antero-ventral region of the *procephalon* at the beginning of stage 15. Fuse at the beginning of stage 16. Contribute to the formation of the *atrium* during stage 16. | 6A ⑤, B, C ③, E ④, F; 7A, C | Adopted from the fruit fly [2,13,14]. |
| *anterior dorsal gap* (transient) | **-** | **-** | **-** | **-** | **V** | **-** | Transversal groove arising between the *procephalon* and the *amnioserosa* during stage 13. Migration starting point for the fused *dorsal folds*. Levels out during stage 15. | 6A ①, C, D | Adopted from the fruit fly [2]. |
| *anterior midgut primordium* (transient) | **-** | **II** | **III** | **IV** | **V** | **-** | Endodermal cell cluster emerging at the anterior tip of the *ventral furrow* at the beginning of stage 7 as part of the *germ layer specification* process. Internalizes at the beginning of stage 8. Fuses with the *posterior midgut primordium* to form the *midgut* during stage 14. | 3C, E, F; 4A ②, F ①; 6A ③, (G) | Adopted from the fruit fly [2,4,15] and the scuttle fly [16]. The terms ‘anterior midgut rudiment’ and ‘anterior midgut anlage’ are used synonymously [2,17]. |
| *atrium* | **-** | **-** | **-** | **-** | **-** | **VI** | Mouth cavity forming anteriorly through dorsal contribution of the *clypeolabrum*, antero-lateral contribution of the *antennomaxillary complexes*, ventrolateral contribution of the *mandibular buds* and ventral contribution of the fused *labial buds* during stage 16 after the *digestive system formation* process has ended. Is connected to the posteriorly located *stomodeum* and resembles the anterior region of the fully developed *digestive tract*. | 7A, C | Adopted from the fruit fly [2,18]. The term ‘cibarium’ is used synonymously [2]. The term ‘atrium’ is occasionally also used for substructures of the heart [19]. |
| *blastoderm nuclei* (transient) | **I** | **-** | **-** | **-** | **-** | **-** | Somatic, superficially located and uniformly distributed nuclei deriving from *zygotic nuclei* at the beginning of stage 4 after the *peripheral migration* process has ended. Constitute together with the *pole buds* the *syncytial blastoderm*. | 2B, D, E; 3A | Established for the medfly [20,21]. Usage similar for the fruit fly [2,22,23]. The term ‘somatic buds’ is used synonymously [2,24]. |
| *cellular blastoderm* (transient) | **-** | **II** | **-** | **-** | **-** | **-** | Uniform superficial cell layer deriving from the *syncytial blastoderm* at the beginning of stage 6 after the *cellularization* process has ended. Envelops the *yolk* *sac.* Gives rise to the *ventral furrow* at the beginning of stage 6 and the *cephalic furrow* during stage 6. Differentiates into the anterior *head*, medio-ventral *thorax*, posterior *abdomen* and the medio-dorsal *amnioserosa* at the beginning of stage 7. | 3A, B, (E, G) | Established for the medfly [20,21,25]. Usage similar for the fruit fly [2,22,26,27]. |
| *cephalic furrow* (transient) | **-** | **II** | **III** | **-** | **-** | **-** | Transversal cleft arising anteromedially in the *cellular blastoderm* during stage 6. Has a slight antero-ventral / postero-dorsal tilt. Deepens at the beginning of stage 7 and separates the anteriorly located *head* from the postero-ventrally located *thorax* and postero-dorsally located *amnioserosa*. Levels out at the beginning of stage 9. | 3B ①, E ①, (F, G); 4A, G | Adopted from the fruit fly [2,4,28,29] and the scuttle fly [5,6]. |
| *clypeolabrum* | **-** | **-** | **-** | **IV** | **V** | **VI** | Large bulge emerging antero-dorsally from the *procephalon* during stage 12. Changes from an antero-dorsal to an antero-ventral orientation at the beginning of stage 14. Retracts posteriorly gets covered by the fused *dorsal folds* during stage 15 as part of the *head involution* process. Contributes to the formation of the *atrium* during stage 16. | 5A, B; 6A, B, C, D, E, F, (G) | Adopted from the fruit fly [2,4,30] and the scuttle fly [5]. |
| *digestive tract* | **-** | **-** | **-** | **-** | **-** | **VI** | Large tubular body captivity originating through consecutive connection of the *atrium*, the *stomodeum*, the *midgut*, the *proctodeum* and the *proctodeal opening* during stage 16 after the *digestive system formation* process has ended. | 7A, C | Adopted from the fruit fly [31,32]. |
| *dorsal epidermal primordia* (transient) | **-** | **-** | **-** | **IV** | **V** | **VI** | Bilateral superficial ectodermal cell layer emerging laterally from the *lateral epidermal primordia* during stage 12. Both flanks migrate dorsolaterally over the *amnioserosa* during stages 13 to 15. Turn into the *dorsal epidermis* at the beginning of stage 16 after the *dorsal zippering* process has ended. | 5A, B ①; 6A, D, G ①②③④; 7A, (C) | Adopted from the fruit fly [2,33]. |
| *dorsal epidermis* | **-** | **-** | **-** | **-** | **-** | **VI** | Large ectodermal cell layer deriving from both flanks of the *dorsal epidermal primordia* at the beginning of stage 16 after the *dorsal zippering* process has ended. | 7A, (C) | Adopted from the fruit fly [2,34,35]. |
| *dorsal folds* (transient) | **-** | **-** | **-** | **-** | **V** | **-** | Protuberance emerging dorsally from the *gnathocephalon* at the anterior region of the *anterior dorsal gap* during stage 13. Migrates over and covers the *procephalon* and *clypeolabrum* during stage 14 as part of the *head involution* process. Turns into the *dorsal pouch* during stage 16 after the *head involution* process has ended. | 6A, B, D, E ⑤; 7C | Adopted from the fruit fly [2,36]. The term ‘dorsal ridge’ is sometimes used synonymously in the fruit fly [2], the scuttle fly [5] and the moth midge [7], sometimes it is used to describes the bilaterally emerging primordia of the fold [2]. |
| *dorsal plate* (transient) | **-** | **II** | **III** | **-** | **-** | **-** | Discoid cell cluster deriving postero-dorsally from the *posterior plate* during stage 7. Carries the *pole cells* at the beginning of stage 8 as part of the *germ cell dynamics* process. Invaginates and turns into the *posterior midgut primordium* during stage 8 as part of the *germ layer specification* process. | 3C ④, (D, E, G), H; 4A, C ①②, H | Adopted from the fruit fly [2,37], the scuttle fly [5] and the moth midge [7]. |
| *dorsal pouch* | **-** | **-** | **-** | **-** | **V** | **VI** | Pocket-like structure deriving antero-dorsally from the fused *dorsal folds* during stage 16 after the *head involution* process has ended. | 7C | Adopted from the fruit fly [2,38]. |
| *ectodermal layer* (transient) | **-** | **II** | **III** | **-** | **-** | **-** | Superficial cell layer differentiating ventrally at the *thorax* and *abdomen* from the *ventral furrow* during stage 7 as part of the *germ layer specification* process. Differentiates into the *amnioproctodeal invagination*, the *ventral epidermal primordium,* and the *lateral epidermal primordium* during stage 8. | 3C, E, F; 4A, (C), G | Adopted from the fruit fly [2]. |
| *embryo* | **-** | **II** | **III** | **IV** | **V** | **VI** | Entirety of the tissue regions differentiating from the *cellular blastoderm* at the beginning of stage 7 that become part of the larva at the end of stage 17. Consists of the *head*, the *thorax,* and the *abdomen*. | 3C, D, E, (G); 5A; 7B, D | Adopted from the fruit fly [2]. |
| *germband* | **-** | **II** | **III** | **IV** | **V** | **VI** | Later on, metameric part of the *embryo*. Composes of the *gnathocephalon*, the *thorax* and the *abdomen*. | 3C, E, F, G; 4A, B, D, E, H, J; 5A; 6A, B, (D) | Adopted from the fruit fly [2,39]. |
| *gnathal protuberances* (transient) | **-** | **-** | **-** | **IV** | **V** | **-** | Small bulges emerging from the *gnathocephalon* during stage 12. Differentiate into the anteriorly located *maxillary buds*, the medially located *mandibular buds* and the posteriorly located *labial buds* during stage 14. | 4B; 6A, C | Adopted from the fruit fly [2,40]. The term ‘gnathal buds’ is used synonymously [2,41]. |
| *gnathocephalon* | **-** | **II** | **III** | **IV** | **V** | **VI** | Later on, metameric subregion forming as the postero-ventral part of the *head* during stage 7. Composes together with the *thorax* and the *abdomen* the *germband* and becomes divided into three segments during stage 11 as part of *metamerization* process. Gives rise to the *gnathal protuberances* during stage 12. | 3C, E, F, G; 4B; (6A, C, D) | Adopted from the fruit fly [2,42]. |
| *head* | **-** | **II** | **III** | **IV** | **V** | **VI** | Large tissue region developing anteriorly from the *cellular blastoderm* at the beginning of stage 7. Constitutes together with the *thorax* and the *abdomen* the *embryo*. Remodels into the non-metameric *procephalon* and the later on metameric *gnathocephalon* during stage 7*.* Internalized during the *head involution* process. | 3C, D, (E), F, (G); 4A, B, J, I; 6A, B, C, D, F; 7A | Adopted from the fruit fly [2,43]. |
| *intersegmental grooves* | **-** | **-** | **-** | **IV** | **V** | **VI** | Transversal grooves arising during stage 12 as part of the *metamerization* process. Separate the *thorax* into three and the *abdomen* into nine segments. Transiently level out during stage 15 and arise again at the beginning of stage 16. | 5A, B ②③④; 6B; 7A, (B), C | Adopted from the fruit fly [2,44], the scuttle fly [5] and the moth midge [7]. The term ‘intersegmental furrows’ is used synonymously [2]. |
| *labial buds* | **-** | **-** | **-** | **-** | **V** | **VI** | Bilateral appendage primordia differentiating from the *gnathal protuberances* during stage 14. Fuse ventrally before retracting during stage 15 as part of the *head involution* process. Contribute to the formation of the *atrium* during stage 16. | 6A ④, 6B, C ②, E ②; (7A, C) | Adopted from the fruit fly [2,45]. |
| *lateral epidermal primordia* (transient) | **-** | **-** | **III** | **IV** | **V** | **VI** | Bilateral superficial ectodermal cell layer differentiating ventrolaterally from the *ectodermal layer* during stage 8. Comprises of tightly packed cells with small nuclei. Give rise to the *dorsal epidermal primordia* during stage 13 and turns into the *lateral epidermis* at the beginning of stage 16. | 4A, (C), G; (5A, B ①); 6A, (D); 7A, (C) | Adopted from the fruit fly [46,47]. The term ‘lateral epidermal anlagen’ is used synonymously [2]. |
| *lateral epidermis* | **-** | **-** | **-** | **-** | **-** | **VI** | Large bilateral ectodermal cell layer deriving from the *lateral epidermal primordia* at the beginning of stage 16. | 7A, (C) | Adopted from the fruit fly [2,48,49]. |
| *mandibular buds* | **-** | **-** | **-** | **-** | **V** | **VI** | Appendage primordia differentiating bilaterally from the *gnathal protuberances* during stage 14. Retract during the *head involution* process. Contribute to the formation of the *atrium* during stage 16. | 6A ②, B, C ①, E ①, F; 7A, C | Adopted from the fruit fly [2]. |
| *maxillary buds* | **-** | **-** | **-** | **-** | **V** | **-** | Bilateral appendage primordia differentiating from the *gnathal protuberances* during stage 14. Fuse with the anteroventrolateral parts of the *procephalon* to form the *antennomaxillary complexes* at the beginning of stage 15. | 6A ⑤, C ③, E ④, F | Adopted from the fruit fly [2,45]. |
| *mesodermal layer* | **-** | **II** | **-** | **-** | **-** | **-** | Internal cell layer differentiating ventrally at the *thorax* and *abdomen* by invagination of cells along the *ventral furrow* during stage 7 as part of the *germ layer specification* process. Involved in mesoderm development (S5 Table). | 3C, E, F | Adopted from the fruit fly [2]. The terms ‘mesodermal primordium’ and ‘mesodermal anlage’ are used synonymously [2]. |
| *midgut* | **-** | **-** | **-** | **-** | **V** | **VI** | Internal endodermal tubular body cavity originating from the fusion of the *anterior midgut primordium* and the *posterior midgut primordium* during stage 14. Is connected to the anteriorly located *stomodeum* and the posterior located *proctodeum* and resembles the medial part of the fully developed *digestive tract*. | 6A ③, (D), (G); (6D); (7A, C) | Adopted from the fruit fly [2,4,17,31,32]. |
| *neuroblasts* | **-** | **-** | **III** | **-** | **-** | **-** | Large nuclei segregating from the *ventral epidermal primordium* at the beginning of stage 9. Involved in neurogenesis (S5 Table). | 4G ①②③ | Adopted from the fruit fly [2,50]. |
| *optic lobe primordium* | **-** | **-** | **III** | **-** | **-** | **-** | Longitudinal cell layer emerging dorsally at the *procephalon* during stage 8. Involved in neurogenesis (S5 Table). | (3D); 4A, J, (I) | Adopted from the fruit fly [2,51]. |
| *pole buds* (transient) | **I** | **-** | **-** | **-** | **-** | **-** | Small bulges emerging at the posterior tip of the *zygote* from the *zygotic nuclei* during stage 3 as part of the *germ cell dynamics* process. Turn into the *pole cells* during stage 4. | 2B, C, F, G | No proper term (‘cytoplasmic protrusions’) in medfly-specific literature [21]. Adopted from the fruit fly [2,52,53] and the scuttle fly [5]. The term ‘polar buds’ is used synonymously [2,52,54]. |
| *pole cells*  *(transient)* | **I** | **II** | **III** | **-** | **-** | **-** | Future germ cells deriving at the posterior tip of the *syncytial blastoderm* from the *pole buds* and undergo two mitotic divisions during stage 4 as part of the *germ cell dynamics* process. Adhere to the *posterior plate*, which forms during stage 6, and to the *dorsal plate*, which forms during stage 7. Internalize during stage 8 after the *germ cell dynamics* process has ended. | 2B ①, F, G; (3C ④, D, E, G, H); (4A, C ①②, H) | Established for the medfly [21]. Usage similar for the fruit fly [2,52,53] and the scuttle fly [5]. |
| *posterior midgut primordium* (transient) | **-** | **-** | **III** | **IV** | **V** | **-** | Endodermal cell cluster deriving from the *dorsal plate* during stage 8 as part of the *germ layer specification* process. Fuses with the *anterior midgut primordium* to form the *midgut* during stage 14 as part of the *digestive system formation* process. | 4A, C ①②, H; 6A ③, (G) | Established for the medfly [21]. Usage similar for the fruit fly [2,3,55] and the scuttle fly [16]. The terms ‘posterior midgut rudiment’ and ‘posterior midgut anlage’ are used synonymously [2,17,21]. |
| *posterior plate* (transient) | **-** | **II** | **-** | **-** | **-** | **-** | Discoid posterior cell cluster to which the *pole cells* adhere emerging at the posterior tip of the *cellular blastoderm* during stage 6. Turns into the *dorsal plate* during stage 7. | 3B, C ④, (D, E, G), H | No proper term (‘blastoderm cell plate’) in medfly-specific literature [21]. Adopted from the fruit fly [2,56] and the moth midge [7]. |
| *procephalon* | **-** | **II** | **III** | **IV** | **V** | **VI** | Non-metameric lobe-like subregion forming as the antero-ventral and dorsal part of the *head* during stage 7. Gives rise to the *clypeolabrum* during stage 12 and partially fuses with the *maxillary buds* to form the *antennomaxillary complexes* during stage 15. | 3C, D, E, F, G; (4A, F); (5A, B);  6A, (B), C, (D), E, F | Adopted from the fruit fly [2,57]. |
| *proctodeal opening* | **-** | **-** | **III** | **IV** | **V** | **VI** | Oval superficial ectodermal opening differentiating at the posterior tip of the germband from the *amnioproctodeal invagination* at the beginning of stage 9 as part of the *digestive system formation* process. Changes from a dorsal to an anterior orientation during stage 9. Flips into the posterior pole during stage 15. Is connected to the anteriorly located *proctodeum* and resembles the posterior part of the fully developed *digestive tract*. | 4A, C, H, (J); (7A, C) | Adopted from the fruit fly [2,58]. |
| *proctodeum* | **-** | **-** | **III** | **IV** | **V** | **VI** | Internal ectodermal tubular body cavity differentiating at the posterior tip of the germband from the *amnioproctodeal invagination* at the beginning of stage 9 as part of the *digestive system formation* process. Becomes internalized during stage 9. Is connected to the anteriorly located *midgut* and the posteriorly located *proctodeal opening* and resembles the posteromedial part of the fully developed *digestive tract*. | 4A, C, H; (7A, C) | Adopted from the fruit fly [2,9,59] and the scuttle fly [60,61]. The term ‘hindgut’ and ‘future hindgut’ are used synonymously [2,62]. |
| *small and large lateral cell stripes* (transient) | **-** | **II** | **-** | **-** | **-** | **-** | Bilateral cell layers undergoing mitotic division at the lateral sides of the *head* at the beginning of stage 7. | 3C ②③, (D), G; (4A) | Convenient term. |
| *stomodeal cell plate* (transient) | **-** | **-** | **III** | **-** | **-** | **-** | Oval cell layer emerging antero-ventrally from the *procephalon* at the beginning of stage 8. Turns into the *stomodeal invagination* during stage 8. | (3E, F); 4A ①, F | Adopted from the fruit fly [2]. The term ‘stomodeal plate’ is used synonymously [4]. |
| *stomodeal invagination* (transient) | **-** | **-** | **III** | **-** | **-** | **-** | Oval superficial ectodermal opening deriving from the *stomodeal cell plate* during stage 8. Turns into the *stomodeum* at the beginning of stage 9. | (3E, F); 4A, F | Adopted from the fruit fly [2,4,63,64], the scuttle fly [5] and the moth midge [7]. |
| *stomodeum* | **-** | **-** | **III** | **IV** | **V** | **VI** | Internal ectodermal tubular body cavity deriving from the *stomodeal invagination* the beginning of stage 9 as part of the *digestive system formation* process. Retracts during stage 14. Is connected to the anteriorly located *atrium* and the posteriorly located *midgut* and resembles the anteromedial part of the fully developed *digestive tract*. | 4A, C, E {3}, F; 6B; (7A, C) | Adopted from the fruit fly [2,4,63,64] and the scuttle fly [5]. The term ‘foregut’ is used synonymously [2,64]. |
| *syncytial blastoderm* (transient) | **I** | **-** | **-** | **-** | **-** | **-** | Uniform superficial nuclei layer deriving from the *zygote* at the beginning of stage 4 after the *peripheral migration* process has ended. Envelops the *yolk*. Undergoes two mitotic division cycles during stage 4. Turns into the *cellular blastoderm* at the beginning of stage 6 after the *cellularization* process has ended. | 2B, (F), (G); 3A | Established for the medfly[20,21,25]. Usage similar for the fruit fly [2,65,66]. |
| *thorax* | **-** | **II** | **III** | **IV** | **V** | **VI** | Large tissue region differentiating medio-ventrally from the *cellular blastoderm* at the beginning of stage 7. Constitutes together with the *head* and the *abdomen* the *embryo*. Composes together with the *gnathocephalon* and the *abdomen* the *germband* and becomes divided into three segments during stage 12 as part of *metamerization* process. | 3C, D, (E, F, G);  (4I, J); 5A, B ②③④; 6A, (D); 7A, C | Adopted from the fruit fly [2,67,68]. |
| *ventral cord* | **-** | **-** | **-** | **-** | **-** | **VI** | Rope ladder-like longitudinal part of the central nervous system becoming visible during stage 16. Spans initially from the third segment of the *thorax* to the 8^th^ segment of the abdomen before shortening posteriorly. | 7A. B, C | Adopted from the fruit fly [2,69]. The term ‘ventral nerve cord’ is used synonymously [2,69–71]. |
| *ventral epidermal primordium*  (transient) | **-** | **-** | **III** | **IV** | **V** | **VI** | Superficial ectodermal cell layer differentiating ventrally from the *ectodermal layer* during stage 8. Comprises of loosely arranged cells with large nuclei. Segregates the *neuroblasts* at the beginning of stage 9. Turns into the *ventral epidermis* at the beginning of stage 16. | 4A, (C), G, (I, J); 6A, (D); 7A, C | Adopted from the fruit fly [2]. The term ‘ventral epidermal anlagen’ is used synonymously [2]. |
| *ventral epidermis* | **-** | **-** | **-** | **-** | **-** | **VI** | Large ectodermal cell layer deriving ventrally from the *ventral epidermal primordium* at the beginning of stage 16 after the *dorsal zippering* process has ended. | 7A, C | Adopted from the fruit fly [2]. |
| *ventral furrow* (transient) | **-** | **II** | **III** | **-** | **-** | **-** | Longitudinal cleft arising ventro-medially in the *cellular blastoderm* at the beginning of stage 6. Involved in mesoderm internalization, differentiation of the *anterior midgut primordium* at the beginning of stage 7 as part of the *germ layer specification* process and the *amnioproctodeal invagination* during stage 8. Continuous closure of the cleft gives rise to the *ectodermal layer* and the *mesodermal layer* at the beginning of stage 8 as part of the *germ layer specification* process. Levels out at the beginning of stage 9. | 3A ①②, E ②③; 3C ①, E ②③, F ①; 4A, H, J | Adopted from the fruit fly [2,72–75], the scuttle fly [5,6] and the moth midge [7]. |
| *ventral mesectoderm* | **-** | **-** | **III** | **-** | **-** | **-** | Longitudinal stripe of mesectodermal cells emerging ventrally along the *thorax* and *abdomen* from the *ventral epidermal primordium* during stage 10. Involved in neurogenesis (**Error! Reference source not found.**). | 4I, (J) | Adopted from the fruit fly [2]. |
| *yolk* (transient) | **I** | **-** | **-** | **-** | **-** | **-** | Initial nutrient reservoir of the egg. Constitutes together with the *zygotic nuclei* the *zygote*. Becomes enveloped by the *syncytial blastoderm* at the beginning of stage 6. Turns into the *yolk sac* at the beginning of stage 6 after the *cellularization* process has ended. | 2A, B, C; 3A | Established for the medfly [20,21]. Usage similar for the fruit fly [2,4]. |
| *yolk sac* (transient) | **-** | **II** | **III** | **IV** | **V** | **-** | Nutrient compartmentation deriving from the *yolk* at the beginning of stage 6 after the *cellularization* process has ended. Migrates to the interior regions and protrudes back to the surface during stages 11 and 12 as part of the *extra-embryonic membrane folding* process. Internalizes together with the *amnioserosa* into the *midgut* during stage 15 after the *extra-embryonic membrane folding* process has ended. | 3A, (C, D, E); 4A, B, H, (I, J); 5A; 6A, D, G | Established for the medfly [20]. Usage similar for the fruit fly [2,76]. |
| *zygote* (transient) | **I** | **-** | **-** | **-** | **-** | **-** | Fertilized egg at the beginning of stage 1. Consists of the *yolk* and the *zygotic nuclei*. Turns into the *syncytial blastoderm* at the beginning of stage 4. | 2B, (C) | Adopted from the fruit fly [2,77]. |
| *zygotic nuclei* (transient) | **I** | **-** | **-** | **-** | **-** | **-** | Internal nuclei resulting from the very first mitotic divisions at the beginning of stage 1. Constitutes together with the *yolk* the *zygote*. Give rise to the *pole buds* during stage 3. Turn into *blastoderm nuclei* at the beginning of stage 4 after the *peripheral migration* process has ended. | 2A ②③, B, (C) | Adopted from the fruit fly [2,78]. |

**References**

1. Rosenberg MI, Lynch JA, Desplan C. Heads and tails: Evolution of antero-posterior patterning in insects. Biochim Biophys Acta. 2009. doi:10.1016/j.bbagrm.2008.09.007

2. Campos-Ortega JA, Hartenstein V. The Embryonic Development of *Drosophila melanogaster*. 2^nd^ Edition. Springer Berlin, Heidelberg. 1997. doi:10.1007/978-3-662-22489-2

3. Reuter R. The gene *serpent* has homeotic properties and specifies endoderm versus ectoderm within the *Drosophila* gut. Development. 1994. doi:10.1242/dev.120.5.1123

4. Kuntz SG, Eisen MB, Lerat E, Vieira C, Carareto C. *Drosophila* Embryogenesis Scales Uniformly across Temperature in Developmentally Diverse Species. PLoS Genet. 2014. doi:10.1371/journal.pgen.1004293

5. Wotton KR, Jiménez-Guri E, García Matheu B, Jaeger J. A Staging Scheme for the Development of the Scuttle Fly *Megaselia abdita*. PLoS One. 2014. doi:10.1371/journal.pone.0084421

6. Caroti F, Urbansky S, Wosch M, Lemke S. Germ line transformation and in vivo labeling of nuclei in Diptera: report on *Megaselia abdita* (Phoridae) and *Chironomus riparius* (Chironomidae). Dev Genes Evol. 2015. doi:10.1007/s00427-015-0504-5

7. Jiménez-Guri E, Wotton KR, Gavilán B, Jaeger J. A Staging Scheme for the Development of the Moth Midge *Clogmia albipunctata*. PLoS One. 2014. doi:10.1371/journal.pone.0084422

8. Colas J-F, Launay J-M, Vonesch J-L, Hickel P, Maroteaux L. Serotonin synchronises convergent extension of ectoderm with morphogenetic gastrulation movements in *Drosophila*. Mech Dev. 1999. doi:10.1016/S0925-4773(99)00141-0

9. Lengyel JA, Iwaki DD. It Takes Guts: The *Drosophila* Hindgut as a Model System for Organogenesis. Dev Biol. 2002. doi:10.1006/dbio.2002.0577

10. Scuderi A, Letsou A. Amnioserosa is required for dorsal closure in *Drosophila*. Dev Dyn. 2005. doi:10.1002/dvdy.20306

11. Lacy ME, Hutson MS. Amnioserosa development and function in *Drosophila* embryogenesis: Critical mechanical roles for an extraembryonic tissue. Dev Dyn. 2016. doi:10.1002/dvdy.24395

12. Schmidt-Ott U. The amnioserosa is an apomorphic character of cyclorrhaphan flies. Dev Genes Evol. 2000. doi:10.1007/s004270050325

13. Higashijima S, Michiue T, Emori Y, Saigo K. Subtype determination of *Drosophila* embryonic external sensory organs by redundant homeo box genes BarH1 and BarH2. Genes Dev. 1992. doi: 10.1101/gad.6.6.1005

14. Fascetti N, Baumgartner S. Expression of *Drosophila* Ten-a, a dimeric receptor during embryonic development. Mech Dev. 2002. doi:10.1016/S0925-4773(02)00055-2

15. Nakagoshi H. Functional specification in the *Drosophila* endoderm. Dev Growth Differ. 2005. doi:10.1111/j.1440-169X.2005.00811.x

16. Rafiqi AM, Lemke S, Schmidt-Ott U. Postgastrular *zen* expression is required to develop distinct amniotic and serosal epithelia in the scuttle fly *Megaselia*. Dev Biol. 2010. doi:10.1016/j.ydbio.2010.01.040

17. Tepass U, Hartenstein V. Epithelium formation in the *Drosophila* midgut depends on the interaction of endoderm and mesoderm. Development. 1994. doi:10.1242/dev.120.3.579

18. Younossi-Hartenstein A, Tepass U, Hartenstein V. Embryonic origin of the imaginal discs of the head of *Drosophila melanogaster*. Roux’s Arch Dev Biol. 1993. doi:10.1007/BF00539891

19. Wu M, Sato TN. On the Mechanics of Cardiac Function of *Drosophila* embryo. PLoS One. 2008. doi:10.1371/journal.pone.0004045

20. Callaini G. Cleavage and membrane formation in the blastoderm of the dipteran *Ceratitis capitata* Wied. J Morphol. 1987. doi:10.1002/jmor.1051930308

21. Riparbelli MG, Callaini G, Dallai R. Primordial germ cell migration in the *Ceratitis capitata* embryo. Tissue Cell. 1996. doi:10.1016/S0040-8166(96)80048-6

22. Mazumdar A, Mazumdar M. How one becomes many: Blastoderm cellularization in *Drosophila melanogaster*. BioEssays. 2002. doi:10.1002/bies.10184

23. Luengo Hendriks CL, Keränen SVE, Fowlkes CC, Simirenko L, Weber GH, DePace AH, et al. Three-dimensional morphology and gene expression in the *Drosophila* blastoderm at cellular resolution I: data acquisition pipeline. Genome Biol. 2006. doi:10.1186/gb-2006-7-12-r123

24. Hunter C, Wieschaus E. Regulated expression of *nullo* is required for the formation of distinct apical and basal adherens junctions in the *Drosophila* blastoderm. J Cell Biol. 2000. doi:10.1083/jcb.150.2.391

25. Schetelig MF, Schmid BGM, Zimowska G, Wimmer EA. Plasticity in mRNA expression and localization of *orthodenticle* within higher Diptera. Evol Dev. 2008. doi:10.1111/j.1525-142X.2008.00283.x

26. Mahowald AP. Electron microscopy of the formation of the cellular blastoderm in *Drosophila melanogaster*. Exp Cell Res. 1963. doi:10.1016/0014-4827(63)90186-1

27. Karr TL, Kornberg TB. *fushi tarazu* protein expression in the cellular blastoderm of *Drosophila* detected using a novel imaging technique. Development. 1989. doi:10.1242/dev.106.1.95

28. Vincent A, Blankenship JT, Wieschaus E. Integration of the head and trunk segmentation systems controls cephalic furrow formation in *Drosophila*. Development. 1997. doi:10.1242/dev.124.19.3747

29. Spencer AK, Siddiqui BA, Thomas JH. Cell shape change and invagination of the cephalic furrow involves reorganization of F-actin. Dev Biol. 2015. doi:10.1016/j.ydbio.2015.03.022

30. Jackson PD, Hoffmann FM. Embryonic expression patterns of the *Drosophila decapentaplegic* gene: Separate regulatory elements control blastoderm expression and lateral ectodermal expression. Dev Dyn. 1994. doi:10.1002/aja.1001990104

31. Buchon N, Osman D, David FPA, Yu Fang H, Boquete J-P, Deplancke B, et al. Morphological and Molecular Characterization of Adult Midgut Compartmentalization in *Drosophila*. Cell Rep. 2013. doi:10.1016/j.celrep.2013.04.001

32. Lemaitre B, Miguel-Aliaga I. The Digestive Tract of *Drosophila melanogaster*. Annu Rev Genet. 2013. doi:10.1146/annurev-genet-111212-133343

33. Fredieu JR, Mahowald AP. Glial interactions with neurons during *Drosophila* embryogenesis. Development. 1989. doi:10.1242/dev.106.4.739

34. Bokor P, DiNardo S. The roles of *hedgehog*, *wingless* and *lines* in patterning the dorsal epidermis in *Drosophila*. Development. 1996. doi:10.1242/dev.122.4.1083

35. Kaltschmidt JA, Lawrence N, Morel V, Balayo T, Fernández BG, Pelissier A, et al. Planar polarity and actin dynamics in the epidermis of *Drosophila*. Nat Cell Biol. 2002. doi:10.1038/ncb882

36. VanHook A, Letsou A. Head involution in *Drosophila*: Genetic and morphogenetic connections to dorsal closure. Dev Dyn. 2008. doi:10.1002/dvdy.21405

37. St Johnston RD, Gelbart WM. *Decapentaplegic* transcripts are localized along the dorsal-ventral axis of the *Drosophila* embryo. EMBO J. 1987. doi:10.1002/j.1460-2075.1987.tb02574.x

38. Zikova M, Da Ponte J-P, Dastugue B, Jagla K. Patterning of the cardiac outflow region in *Drosophila*. Proc Natl Acad Sci U S A. 2003. doi:10.1073/pnas.2133156100

39. Irvine KD, Wieschaus E. Cell intercalation during *Drosophila* germband extension and its regulation by pair-rule segmentation genes. Development. 1994. doi:10.1242/dev.128.18.3445

40. Davis GK, Jaramillo CA, Patel NH. Pax group III genes and the evolution of insect pair-rule patterning. Development. 2001. doi:10.1242/dev.128.18.3445

41. Cho NK, Keyes L, Johnson E, Heller J, Ryner L, Karim F, et al. Developmental control of blood cell migration by the *Drosophila* VEGF pathway. Cell. 2002. doi:10.1016/s0092-8674(02)00676-1

42. Lynch JA, El-Sherif E, Brown SJ. Comparisons of the embryonic development of *Drosophila*, *Nasonia*, and *Tribolium*. Wiley Interdiscip Rev Dev Biol. 2012. doi:10.1002/wdev.3

43. Schmidt-Ott U, González-Gaitán M, Jäckle H, Technau GM. Number, identity, and sequence of the *Drosophila* head segments as revealed by neural elements and their deletion patterns in mutants. Proc Natl Acad Sci U S A. 1994. doi:10.1073/pnas.91.18.8363

44. Liu X, Lengyel JA. Drosophila arc Encodes a Novel Adherens Junction-Associated PDZ Domain Protein Required for Wing and Eye Development. Dev Biol. 2000. doi:10.1006/dbio.2000.9689

45. Pultz MA, Diederich RJ, Cribbs DL, Kaufman TC. The *proboscipedia* locus of the Antennapedia complex: a molecular and genetic analysis. Genes Dev. 1988. doi:10.1101/gad.2.7.901

46. Su YC, Treisman JE, Skolnik EY. The *Drosophila* Ste20-related kinase misshapen is required for embryonic dorsal closure and acts through a JNK MAPK module on an evolutionarily conserved signaling pathway. Genes Dev. 1998. doi:10.1101/gad.12.15.2371

47. Kyriakis JM. Signaling by the germinal center kinase family of protein kinases. J Biol Chem. 1999. doi:10.1074/jbc.274.9.5259

48. Bejsovec A, Martinez Arias A. Roles of wingless in patterning the larval epidermis of *Drosophila*. Development. 1991. doi:10.1242/dev.113.2.471

49. Fernandez R, Takahashi F, Liu Z, Steward R, Stein D, Stanley ER. The *Drosophila* shark tyrosine kinase is required for embryonic dorsal closure. Genes Dev. 2000. doi:10.1101/gad.14.5.604

50. Knoblich JA, Schober M, Schaefer M. Bazooka recruits Inscuteable to orient asymmetric cell divisions in *Drosophila* neuroblasts. Nature. 1999. doi:10.1038/990135

51. Lee KJ, Mukhopadhyay M, Pelka P, Campos AR, Steller H. Autoregulation of the *Drosophila disconnected* Gene in the Developing Visual System. Dev Biol. 1999. doi:10.1006/dbio.1999.9420

52. Deshpande G, Calhoun G, Schedl P. The *Drosophila* fragile X Protein dFMR1 is Required During Early Embryogenesis for Pole Cell Formation and Rapid Nuclear Division Cycles. Genetics. 2006. doi:10.1534/genetics.106.062414

53. Dansereau DA, Lasko P. The Development of Germline Stem Cells in *Drosophila*. Methods Mol Biol. 2008. doi:10.1007/978-1-60327-214-8_1

54. Deshpande G, Calhoun G, Schedl P. *Drosophila* argonaute-2 is required early in embryogenesis for the assembly of centric/centromeric heterochromatin, nuclear division, nuclear migration, and germ-cell formation. Genes Dev. 2005. doi:10.1101/gad.1316805

55. Hozumi S, Maeda R, Taniguchi K, Kanai M, Shirakabe S, Sasamura T, et al. An unconventional myosin in *Drosophila* reverses the default handedness in visceral organs. Nature. 2006. doi:10.1038/nature04625

56. Beller M, Blanke S, Brentrup D, Jäckle H. Identification and expression of Ima, a novel Ral-interacting *Drosophila* protein. Mech Dev. 2002. doi:10.1016/S0925-4773(03)00125-4

57. de Velasco B, Mandal L, Mkrtchyan M, Hartenstein V. Subdivision and developmental fate of the head mesoderm in *Drosophila melanogaster*. Dev Genes Evol. 2006. doi:10.1007/s00427-005-0029-4

58. Kuziora MA, McGinnis W. Different transcripts of the *Drosophila* Abd-B gene correlate with distinct genetic sub-functions. EMBO J. 1988. doi:10.1002/j.1460-2075.1988.tb03190.x

59. Murakami R, Shigenaga A, Kawakita M, Takimoto K, Yamaoka I, Akasaka K, et al. *aproctous*, a locus that is necessary for the development of the proctodeum in *Drosophila* embryos, encodes a homolog of the vertebrate *Brachyury* gene. Roux’s Arch Dev Biol. 1995. doi:10.1007/BF00188847

60. Kwan CW, Gavin-Smyth J, Ferguson EL, Schmidt-Ott U. Functional evolution of a morphogenetic gradient. Elife. 2016. doi:10.7554/eLife.20894

61. Rafiqi AM, Lemke S, Ferguson S, Stauber M, Schmidt-Ott U. Evolutionary origin of the amnioserosa in cyclorrhaphan flies correlates with spatial and temporal expression changes of *zen*. Proc Natl Acad Sci U S A. 2008. doi:10.1073/pnas.0709145105

62. Takashima S, Paul M, Aghajanian P, Younossi-Hartenstein A, Hartenstein V. Migration of *Drosophila* intestinal stem cells across organ boundaries. Development. 2013. doi:10.1242/dev.082933

63. González-Gaitán M, Jäckle H. Invagination centers within the *Drosophila* stomatogastric nervous system anlage are positioned by *Notch*-mediated signaling which is spatially controlled through *wingless*. Development. 1995. doi:10.1242/dev.121.8.2313

64. Farge E. Mechanical Induction of Twist in the *Drosophila* Foregut/Stomodeal Primordium. Curr Biol. 2003. doi:10.1016/S0960-9822(03)00576-1

65. Sherlekar A, Rikhy R. *Drosophila* embryo syncytial blastoderm cellular architecture and morphogen gradient dynamics: Is there a correlation? Front Biol (Beijing). 2012. doi:10.1007/s11515-011-1160-4

66. Ali-Murthy Z, Kornberg TB, Schneider M, Frei E, Noll M, Baumgartner S, et al. Bicoid gradient formation and function in the *Drosophila* pre-syncytial blastoderm. Elife. 2016. doi:10.7554/eLife.13222

67. Struhl G. Genes controlling segmental specification in the *Drosophila* thorax. Proc Natl Acad Sci U S A. 1982. doi:10.1073/pnas.79.23.7380

68. Peña-Rangel MT, Rodriguez I, Riesgo-Escovar JR. A misexpression study examining dorsal thorax formation in *Drosophila melanogaster*. Genetics. 2002. doi:10.1093/genetics/160.3.1035

69. Cardona A, Larsen C, Hartenstein V. Neuronal fiber tracts connecting the brain and ventral nerve cord of the early *Drosophila* larva. J Comp Neurol. 2009. doi:10.1002/cne.22086

70. Boerner J, Duch C. An average shape standard atlas for the adult *Drosophila* ventral nerve cord. J Comp Neurol. 2010. doi:10.1002/cne.22346

71. Gan G, Lv H, Xie W, Technau G, Buxton E. Morphological Identification and Development of Neurite in *Drosophila* Ventral Nerve Cord Neuropil. PLoS One. 2014. doi:10.1371/journal.pone.0105497

72. Sweeton D, Parks S, Costa M, Wieschaus E. Gastrulation in *Drosophila*: the formation of the ventral furrow and posterior midgut invaginations. Development. 1991. doi:10.1242/dev.112.3.775

73. Conte V, Ulrich F, Baum B, Muñoz J, Veldhuis J, Brodland W, et al. A Biomechanical Analysis of Ventral Furrow Formation in the *Drosophila melanogaster* Embryo. PLoS One. 2012. doi:10.1371/journal.pone.0034473

74. Spahn P, Reuter R, Herz H, Bartoszewski S, Schnorrer F. A Vertex Model of *Drosophila* Ventral Furrow Formation. PLoS One. 2013. doi:10.1371/journal.pone.0075051

75. Polyakov O, He B, Swan M, Shaevitz JW, Kaschube M, Wieschaus E. Passive mechanical forces control cell-shape change during *Drosophila* ventral furrow formation. Biophys J. 2014. doi:10.1016/j.bpj.2014.07.013

76. Schöck F, Perrimon N. Retraction of the *Drosophila* germ band requires cell-matrix interaction. Genes Dev. 2003. doi:10.1101/gad.1068403

77. Blachon S, Khire A, Avidor-Reiss T. The origin of the second centriole in the zygote of *Drosophila melanogaster*. Genetics. 2014. doi:10.1534/genetics.113.160523

78. Ali-Murthy Z, Lott SE, Eisen MB, Kornberg TB, Ruan J. An Essential Role for Zygotic Expression in the Pre-Cellular *Drosophila* Embryo. PLoS Genet. 2013. doi:10.1371/journal.pgen.1003428
