# Supplementary material for: A two-level staging system for the embryonic morphogenesis of the Mediterranean fruit fly (medfly) Ceratitis capitata
Source: PLoS One. 2024 Dec 30;19(12):e0316391. doi: 10.1371/journal.pone.0316391 (PMC11684674; doi:10.1371/journal.pone.0316391)
Supplement: S5 Table — Our study characterizes the embryogenesis of the medfly mainly on the tissue and cell levels. Thus, we do not describe any subcellular structures and processes with the exception of nuclei. (DOCX) [file pone.0316391.s005.docx]

**S5 Table**

| **structure / process** | **rationale** |
| --- | --- |
| anterior transversal furrow | Does not arise during medfly embryogenesis (see *extra-embryonic membrane folding* in S3 Table). |
| apoptosis | Not properly detectable with our method. |
| cell membranes | Not properly detectable when using the TREhs43-hidAla5_F1m2 transgenic line. For completeness, cell membranes are mentioned in the description of blastoderm formation and early gastrulation (I-5 and II-6) based on the assumption that they form after the 12^th^ synchronous division wave similar to *Drosophila melanogaster*. |
| epiphysis | Not properly identifiable in our data. |
| fat bodies | Not properly identifiable in our data. |
| gastric caeca | Not properly identifiable in our data. |
| gonads | Not properly identifiable in our data. |
| macrophages | Not properly detectable with our method. |
| Malpighian tubes | Not properly identifiable in our data. |
| mesoderm development | Only differentiation of the *ventral furrow* into the *mesodermal layer* and the emergence of the *ventral mesectoderm*, which gives rise to the mesectoderm, can be properly observed in our data. Thus, any further mesoderm development is not described within this study (see *germ layer specification* in S3 Table). |
| mitotic divisions | Only mitotic divisions during blastoderm formation (stages I-1 to I-5) and early gastrulation (stages II-6 and II-7) can be properly identified in our data. Thus, any further mitotic division is not described within this study. |
| neurogenesis | Only segregation of *neuroblasts* from the *ventral epidermal primordium*, emergence and mitotic division of the *optic lobe primordium* and shortening of the posterior tip of the *ventral cord* can be properly observed in our data. Thus, any further neuronal development is not described within this study. |
| esophagus | In our study, the *stomodeum* is not further subdivided into pharynx, esophagus and proventriculus. |
| oocyte nucleus | Not properly identifiable in our data. The first nuclei that we describe are the *zygotic nuclei*. |
| parasegmental furrows | Not properly detectable in our data. If any parasegmental furrows arise in the medfly, they are most probably very faint. |
| pharynx | In our study, the *stomodeum* is not further subdivided into pharynx, esophagus and proventriculus. |
| posterior transversal furrow | Does not arise during medfly embryogenesis (see *extra-embryonic membrane folding* in S3 Table). |
| proventriculus | In our study, the *stomodeum* is not further subdivided into pharynx, esophagus and proventriculus. |
| salivary glands | No substructures (placodes, lobes, ducts, tubes) properly identifiable in our data. |
| spiracles | Not properly identifiable in our data. |
| tentorium | Not properly identifiable in our data. |
| tracheal system | No substructures (placodes, pits, tubules) properly identifiable in our data. |
| vitellophages | Not properly detectable with our method. |
| yolk granules | Not properly detectable with our method. |
