# Supplementary figures and images for: A two-level staging system for the embryonic morphogenesis of the Mediterranean fruit fly (medfly) Ceratitis capitata
Source: PLoS One. 2024 Dec 30;19(12):e0316391. doi: 10.1371/journal.pone.0316391 (PMC11684674; doi:10.1371/journal.pone.0316391)

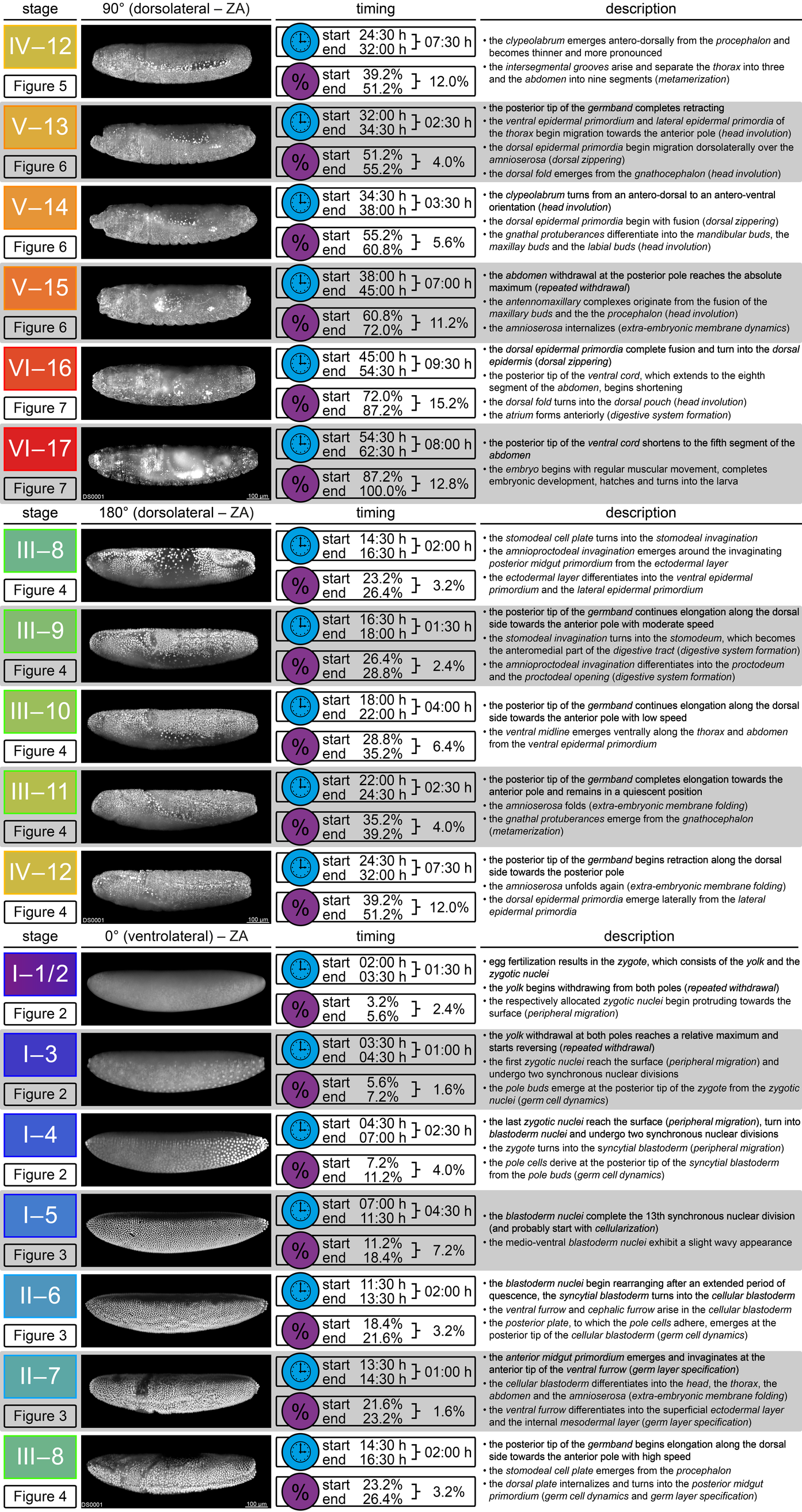

Supplement: S1 Fig — The first column indicates embryonic events (Roman numerals), stages (Arabic numerals) and reference to the corresponding figures. The second column shows exemplary Z maximum projections of embryos in a suitable orientation at the given stage. The third column summarizes start, duration, and end of the given stage in hours (blue) and in percent of total development (purple). The fourth column outlines up to four developmental actions during the respective stage. ZA, Z maximum projection with image adjustment. (TIF) [file pone.0316391.s006.tif]
